# Supplementary material for: BIBR1532 inhibits proliferation and enhances apoptosis in multiple myeloma cells by reducing telomerase activity
Source: PeerJ. 2023 Nov 8;11:e16404. doi: 10.7717/peerj.16404 (PMC10638922; doi:10.7717/peerj.16404)

Figure 3B

The original blot of GAPDH in K562 cells

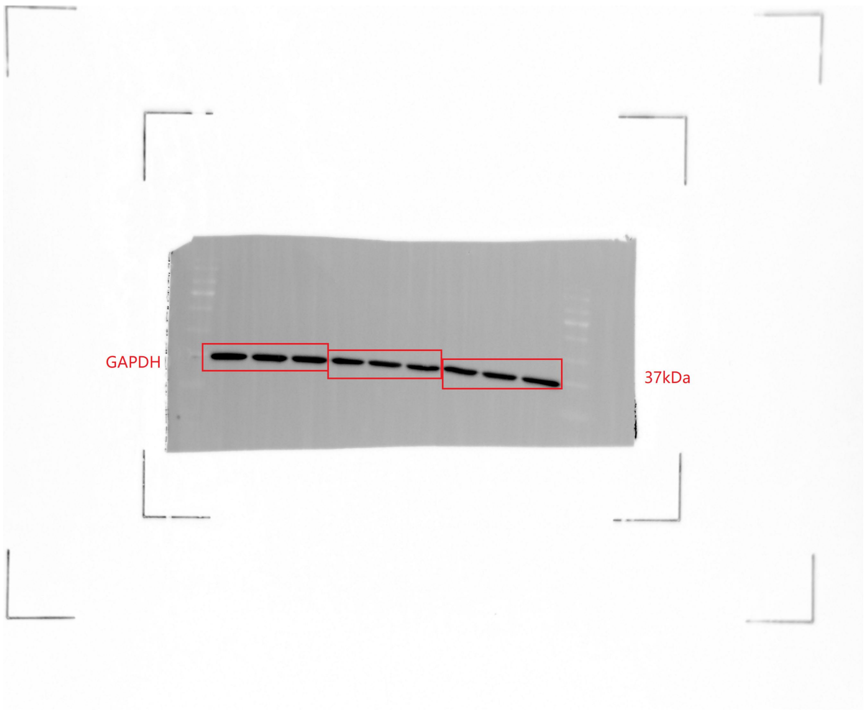

Figure 3B
The original blot of Bcl-XL in K562 cells

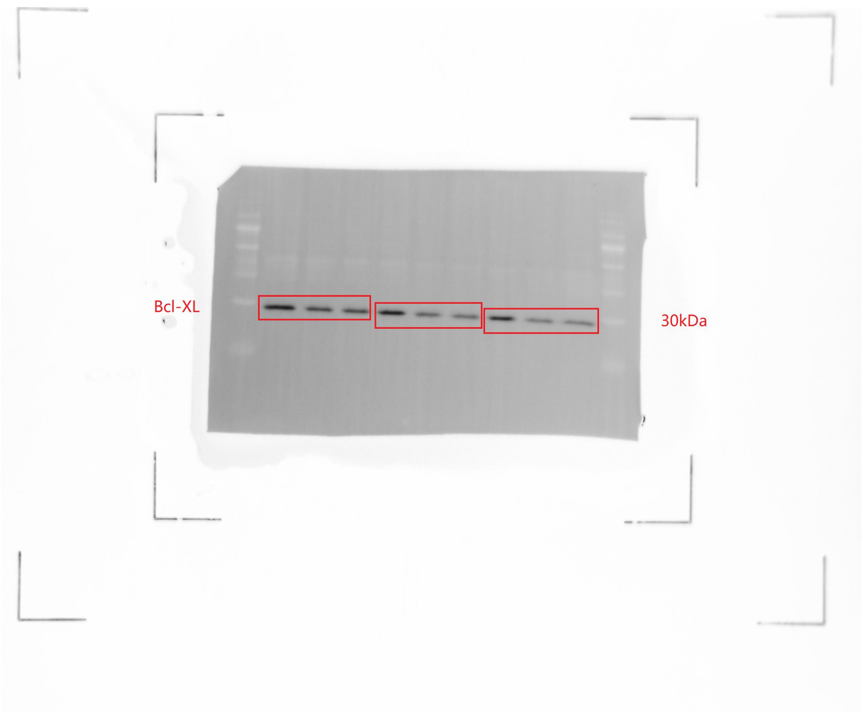

Figure 3B

The original blot of Bad in K562 cells

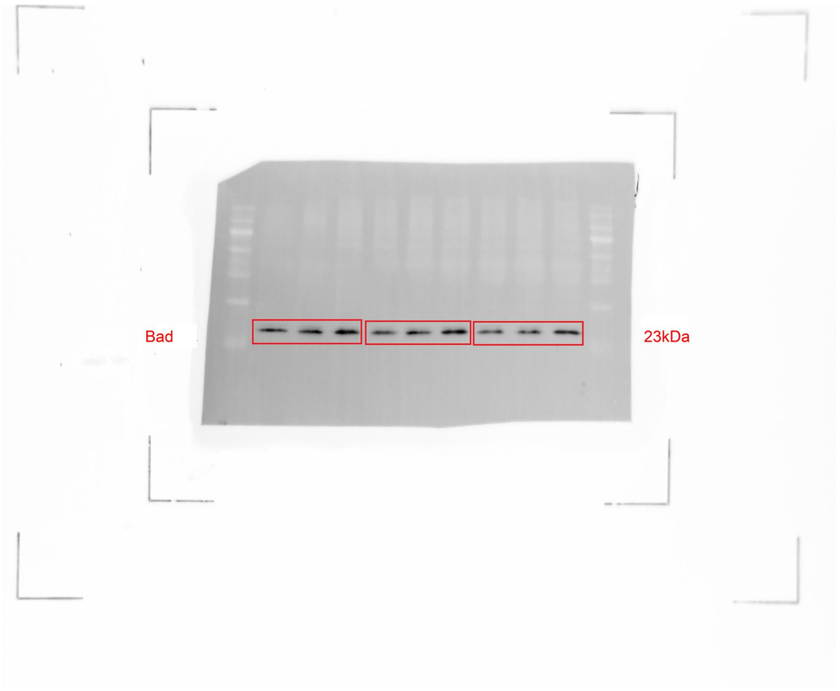

Figure 3B

The original blot of Survivin in K562 cells

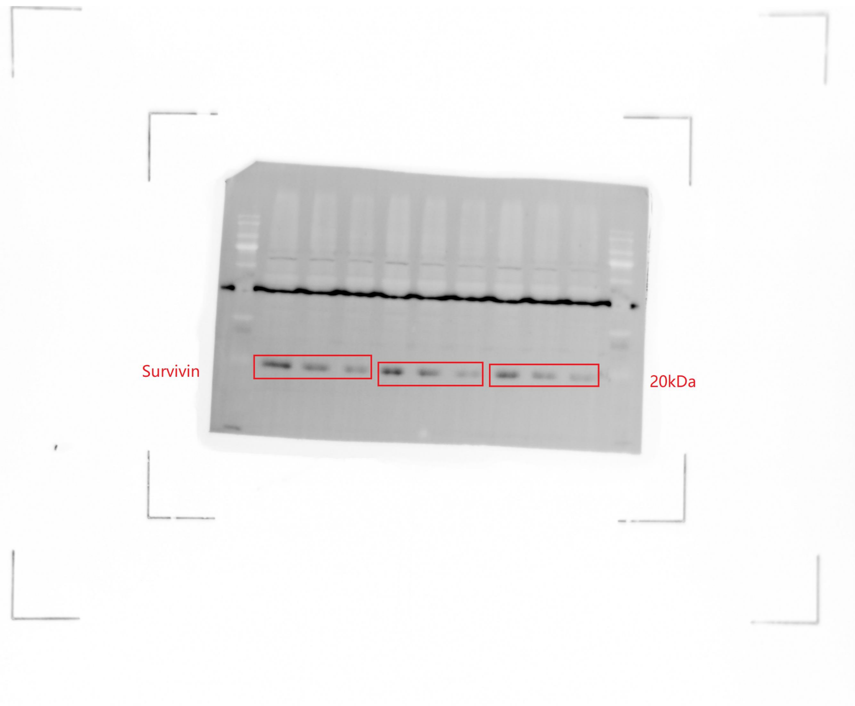

Figure 3B

The original blot of GAPDH in MEG-01 cells

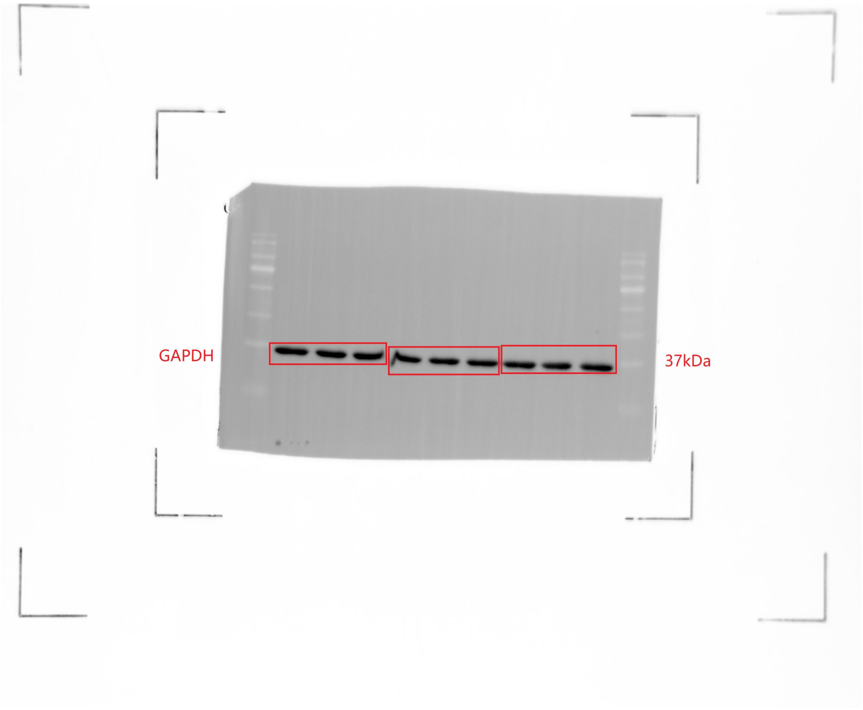

Figure 3B

The original blot of Bcl-XL in MEG-01 cells

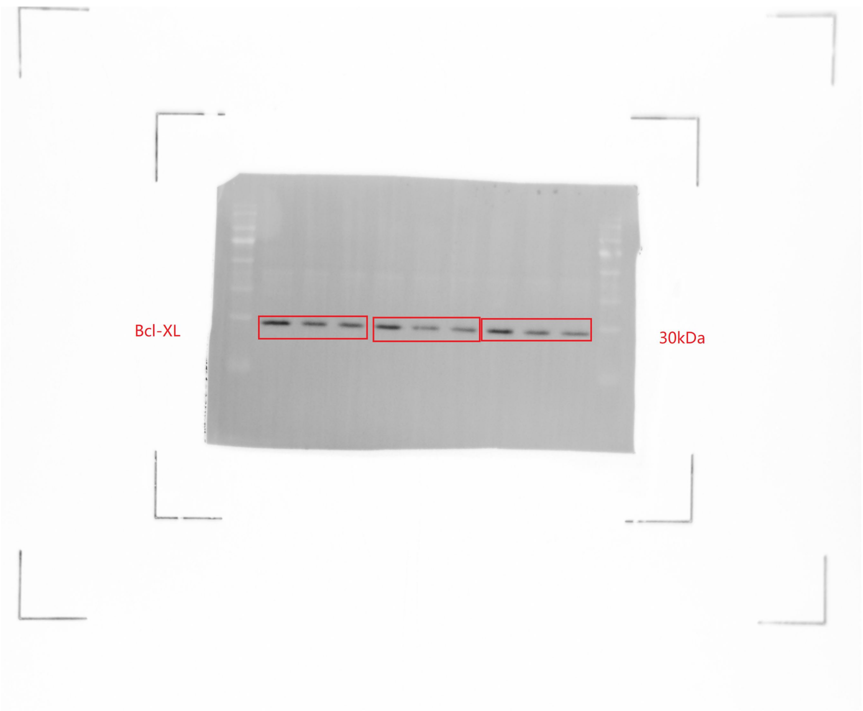

Figure 3B
The original blot of Bad in MEG-01 cells

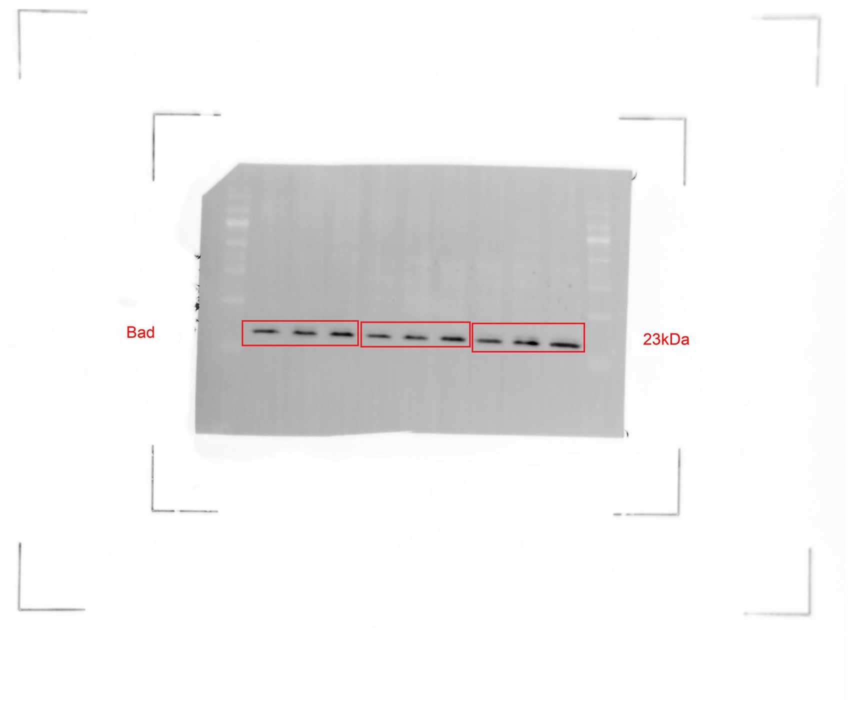

Figure 3B

The original blot of Survivin in MEG-01 cells

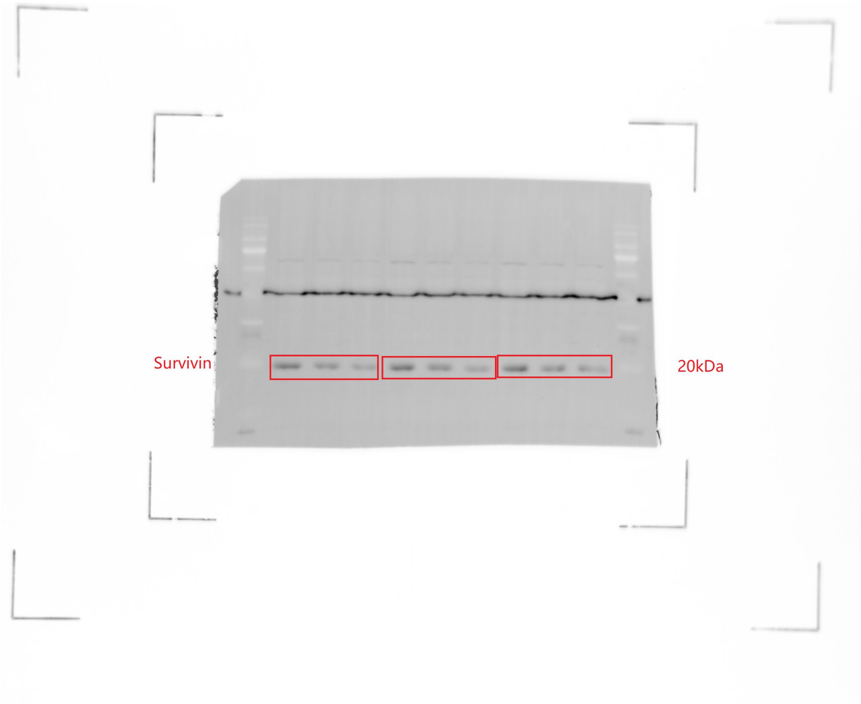

Figure 4B

The original blot of GAPDH in K562 cells

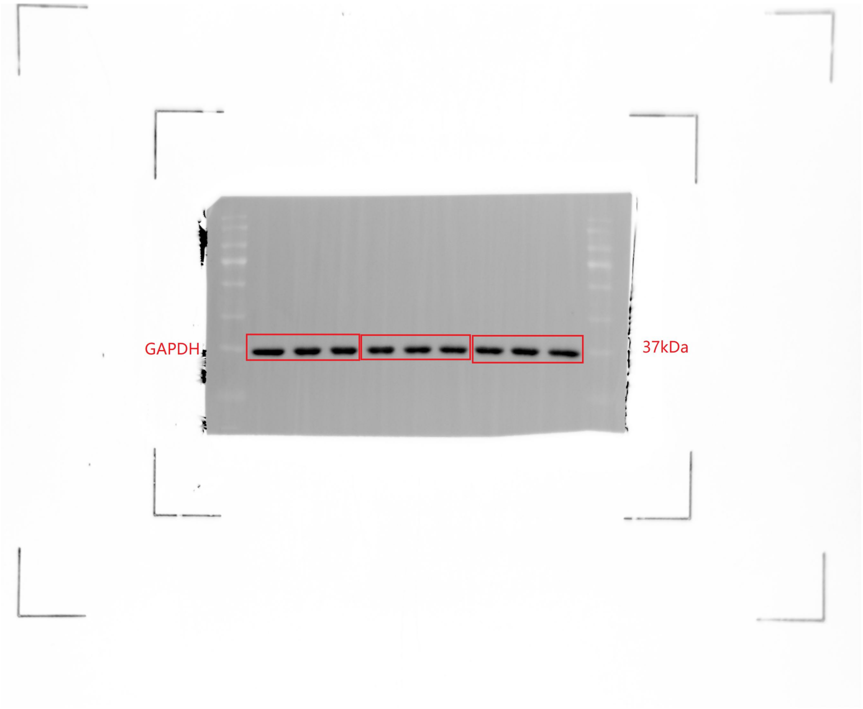

Figure 4B

The original blot of hTERT in K562 cells

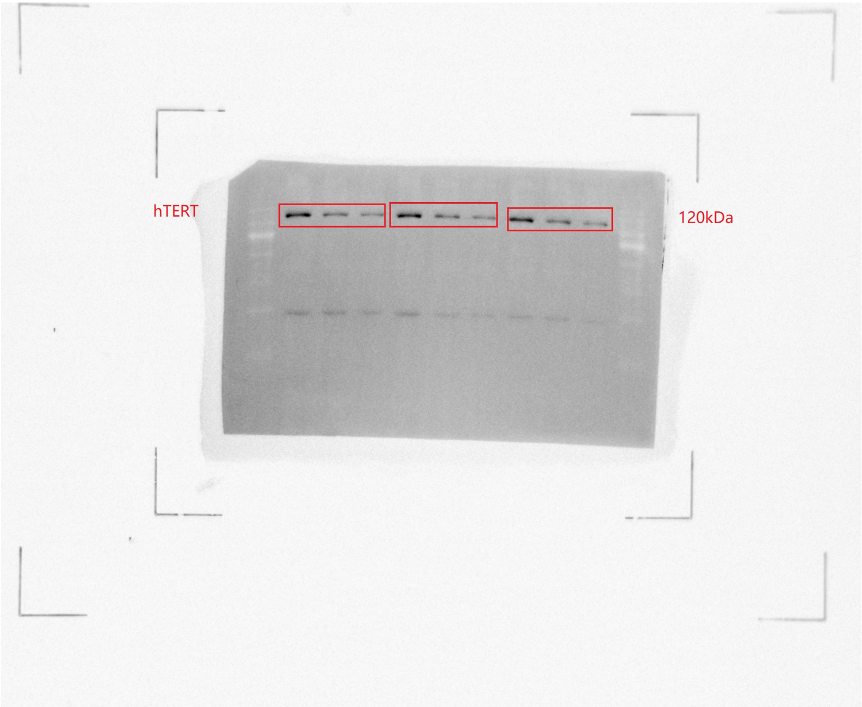

Figure 4B

The original blot of c-MYC in K562 cells

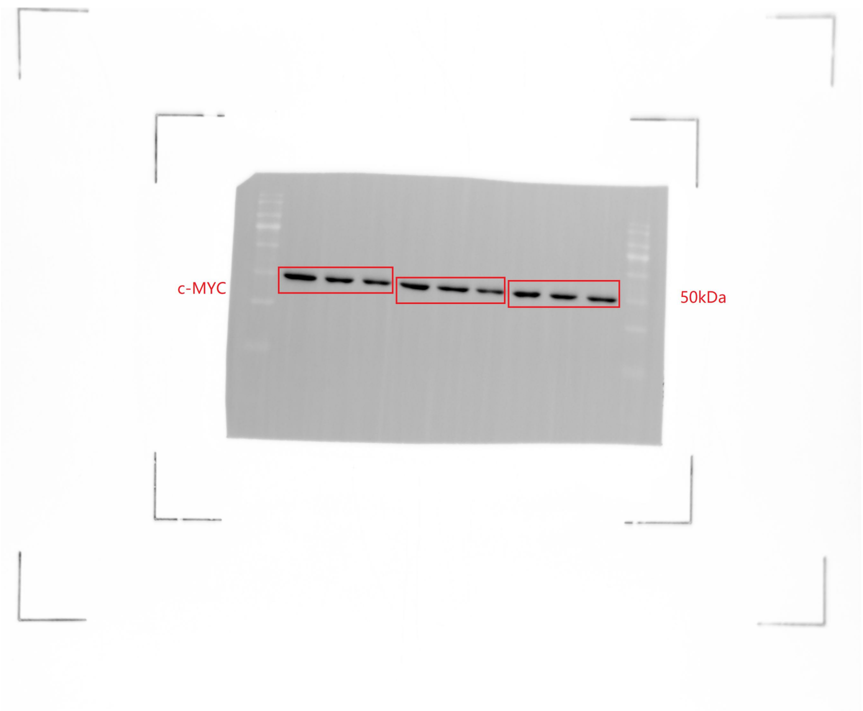

Figure 4B

The original blot of GAPDH in MEG-01 cells

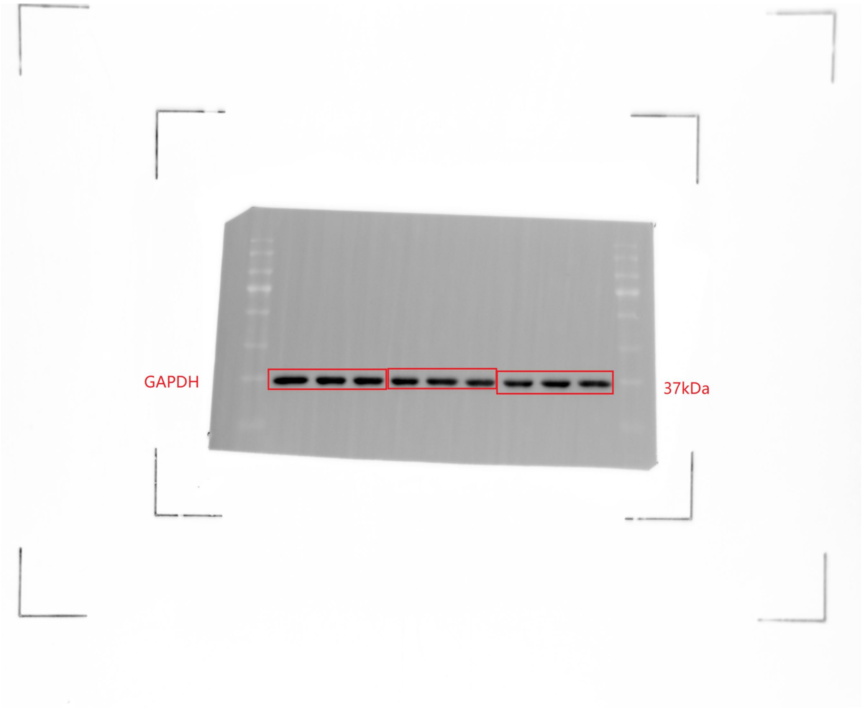

Figure 4B

The original blot of hTERT in MEG-01 cells

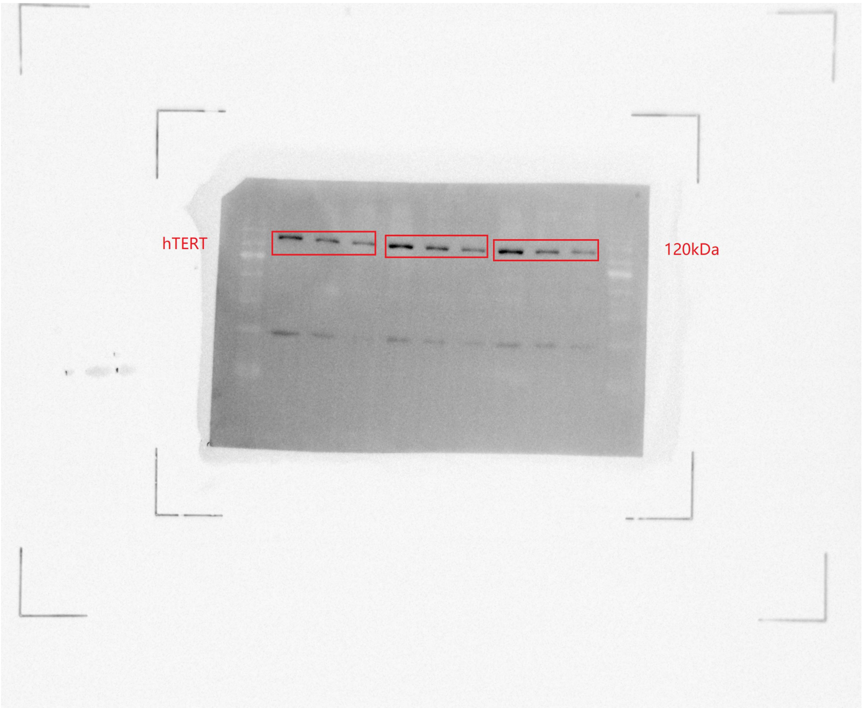

Figure 4B

The original blot of c-MYC in MEG-01 cells

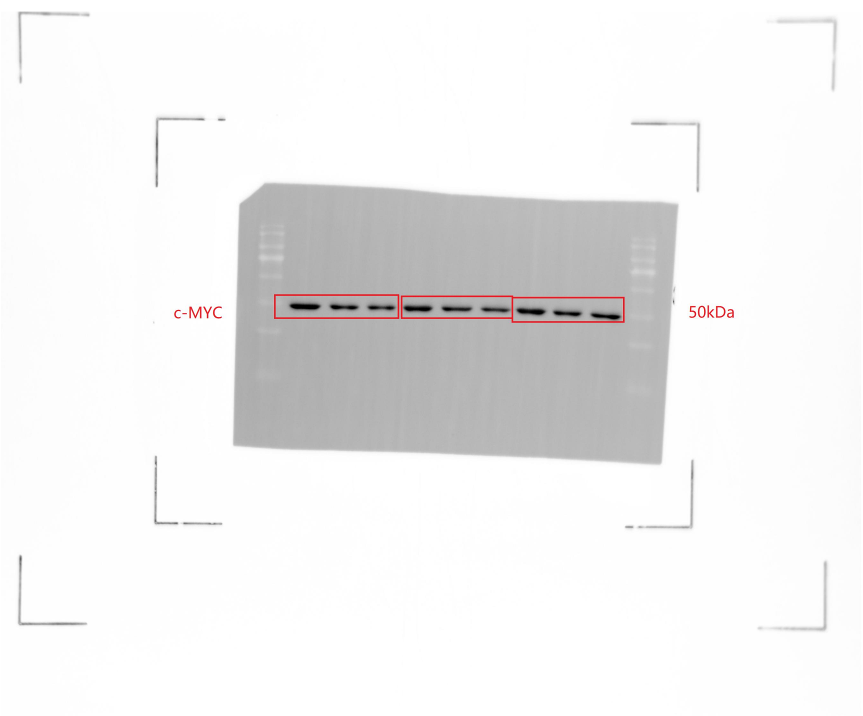

Figure 5A

The original blot of GAPDH in K562 cells

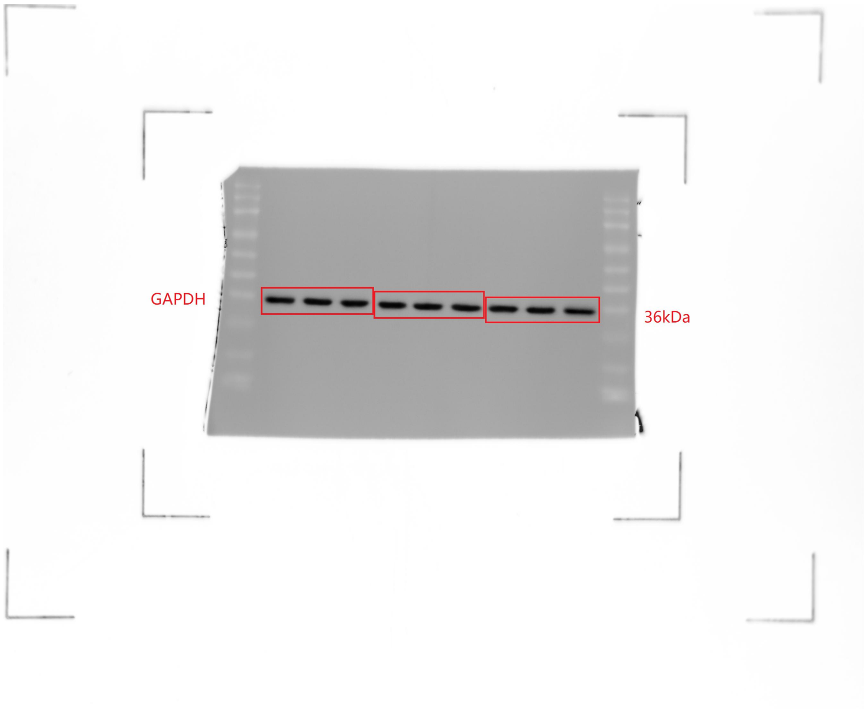

Figure 5A

The original blot of PI3K in K562 cells

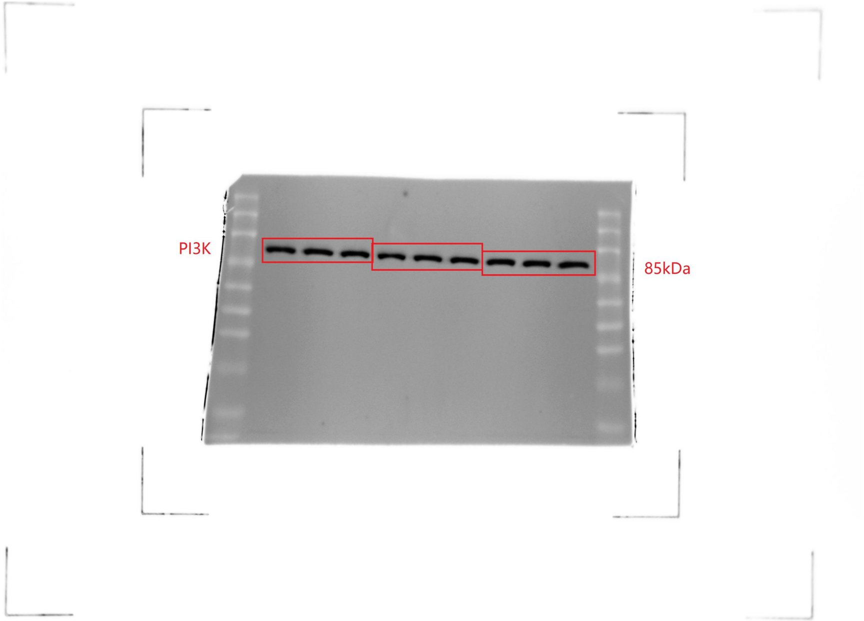

Figure 5A

The original blot of p-PI3K in K562 cells

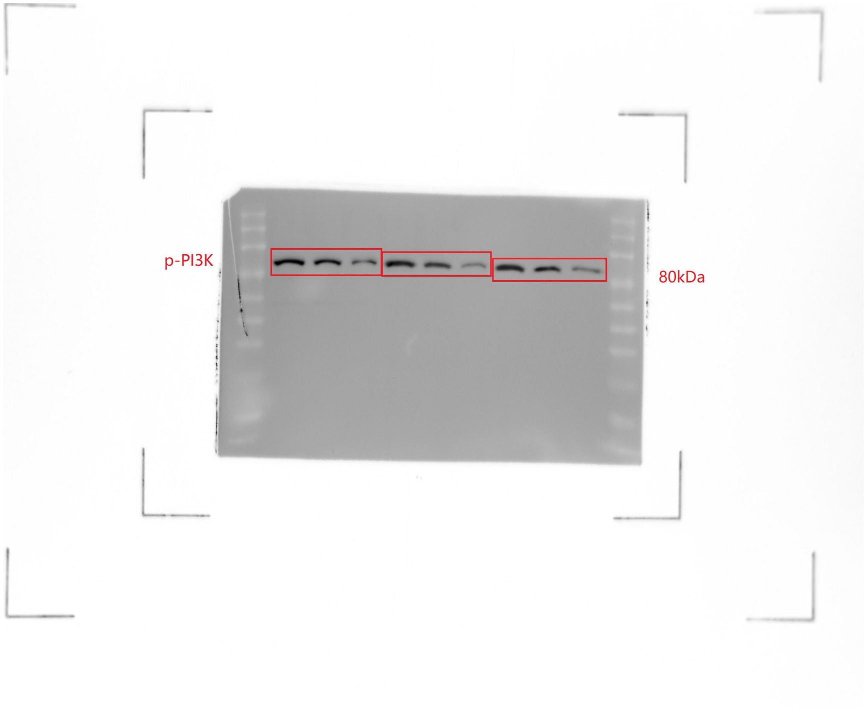

Figure 5A

The original blot of GAPDH in K562 cells

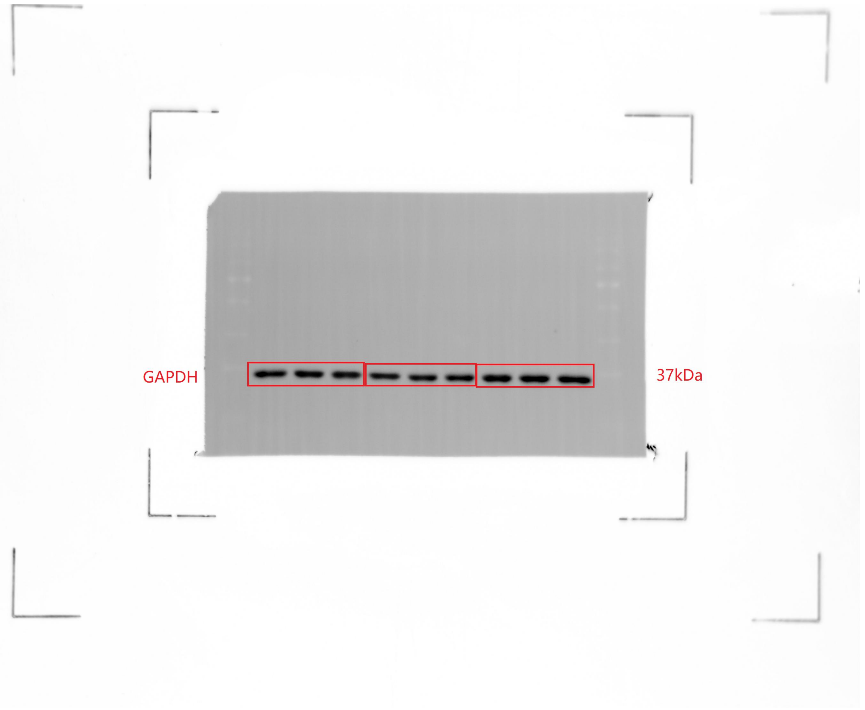

Figure 5A

The original blot of AKT in K562 cells

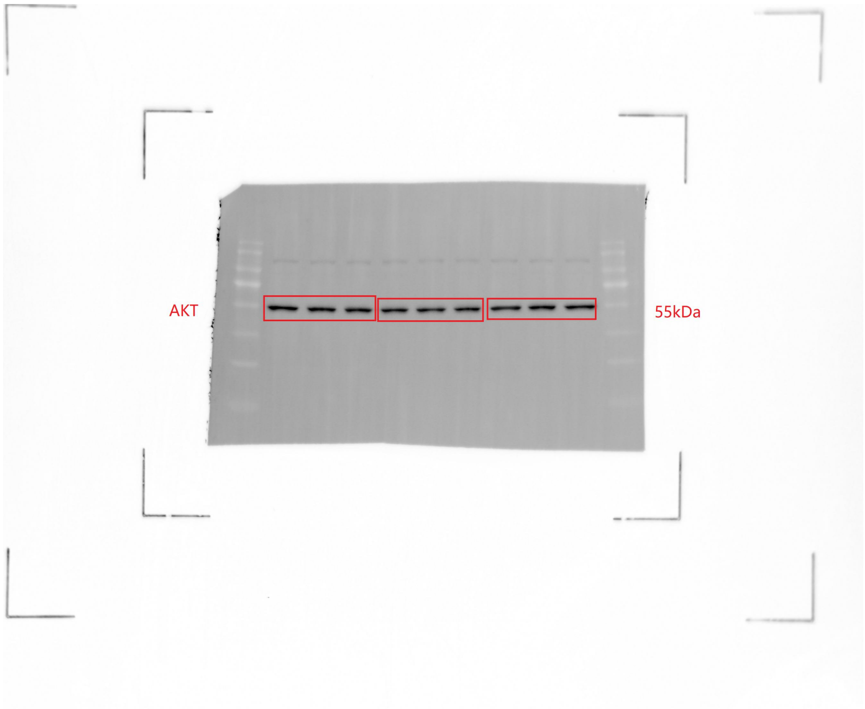

Figure 5A

The original blot of P-AKT in K562 cells

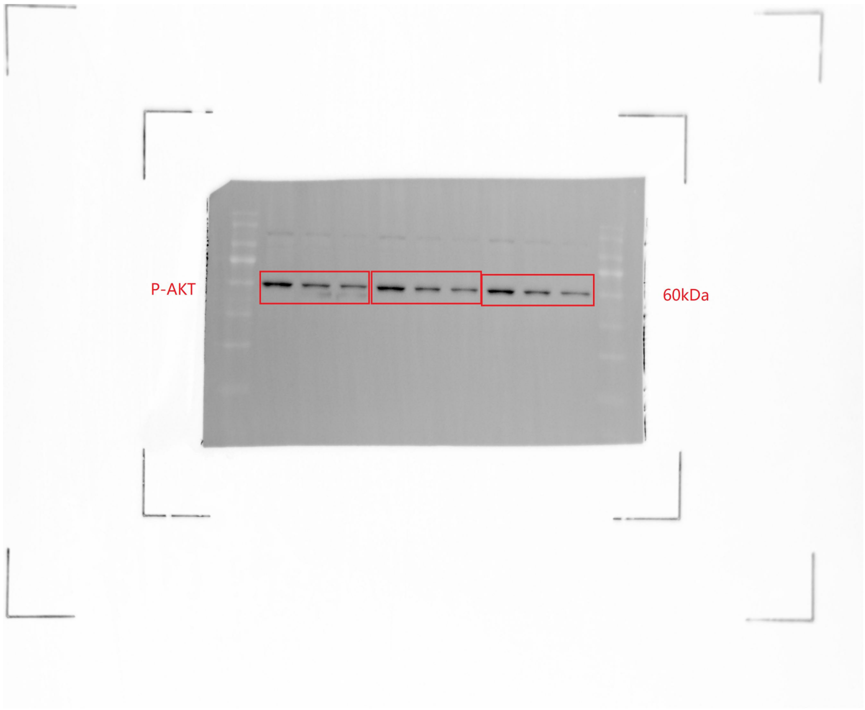

Figure 5A

The original blot of mTOR in K562 cells

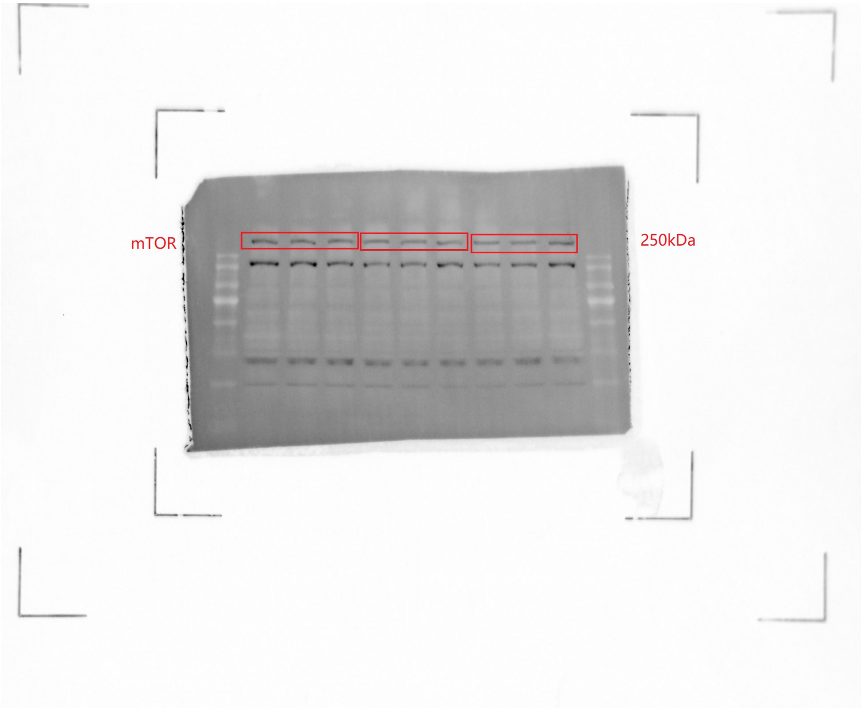

Figure 5A

The original blot of P-mTOR in K562 cells

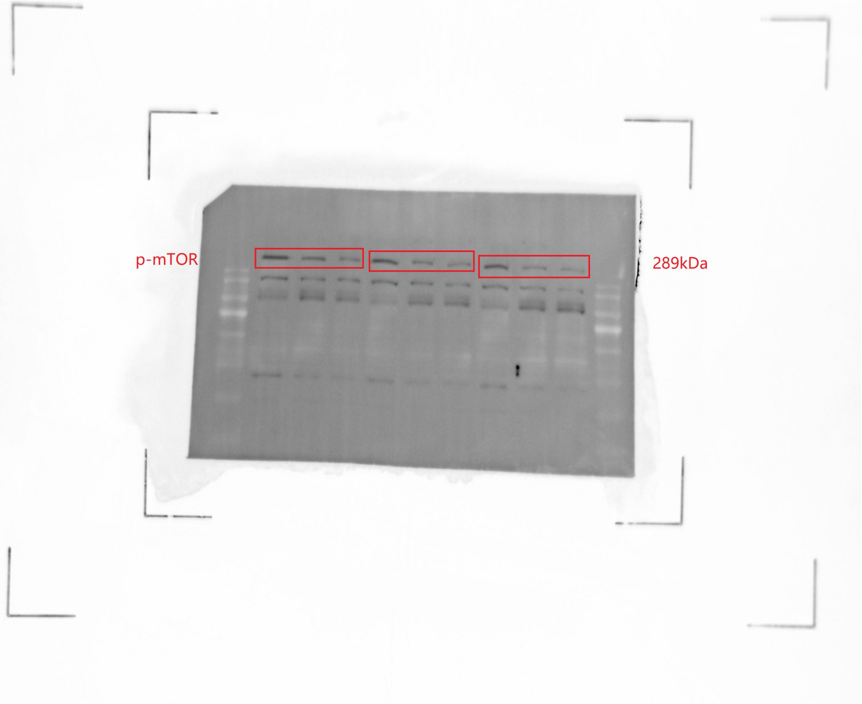

Figure 5A

The original blot of ERK in K562 cells

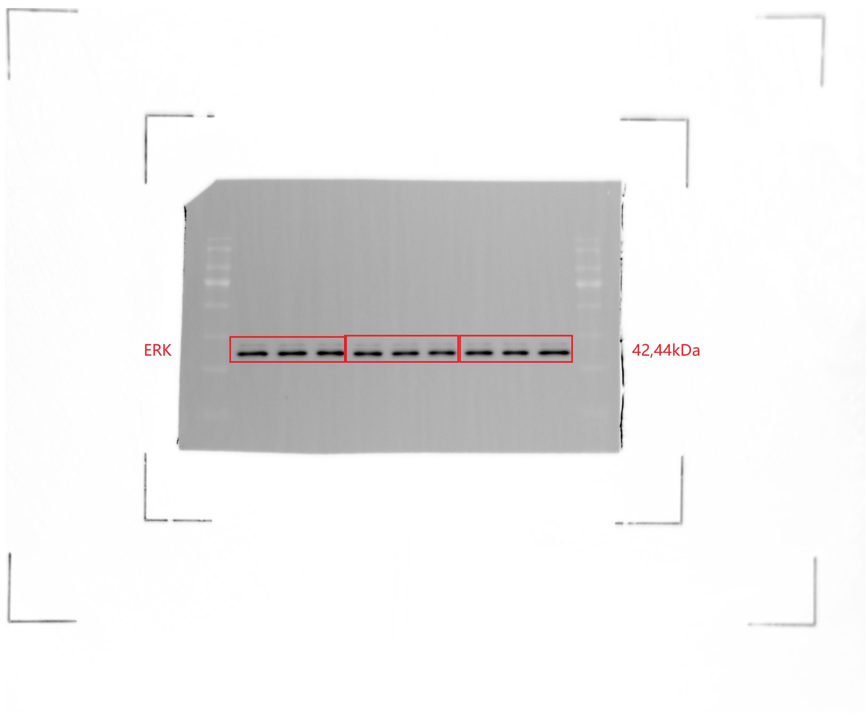

Figure 5A
The original blot of P-ERK in K562 cells

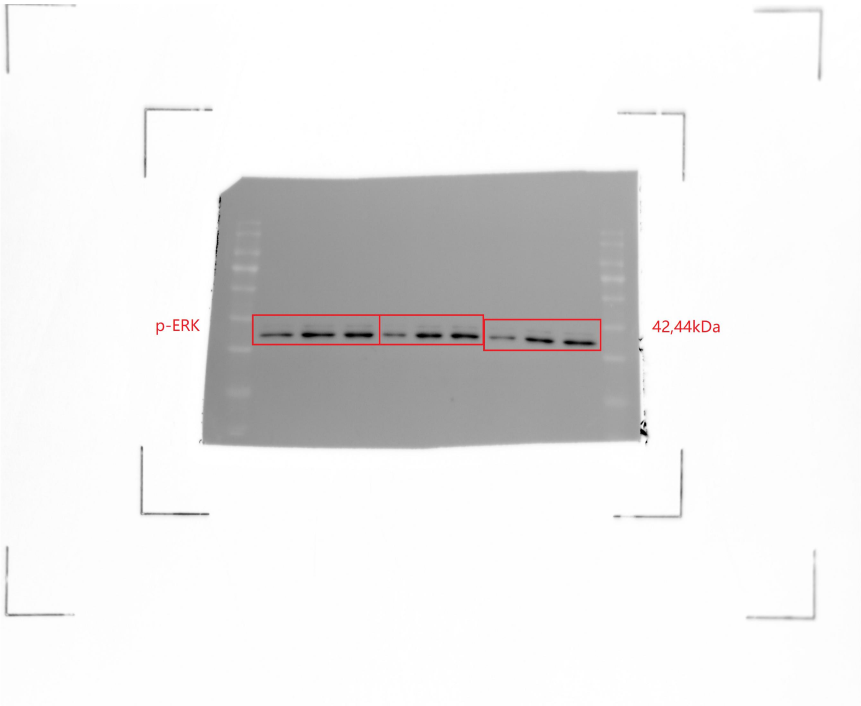

Figure 5A

The original blot of MAPK in K562 cells

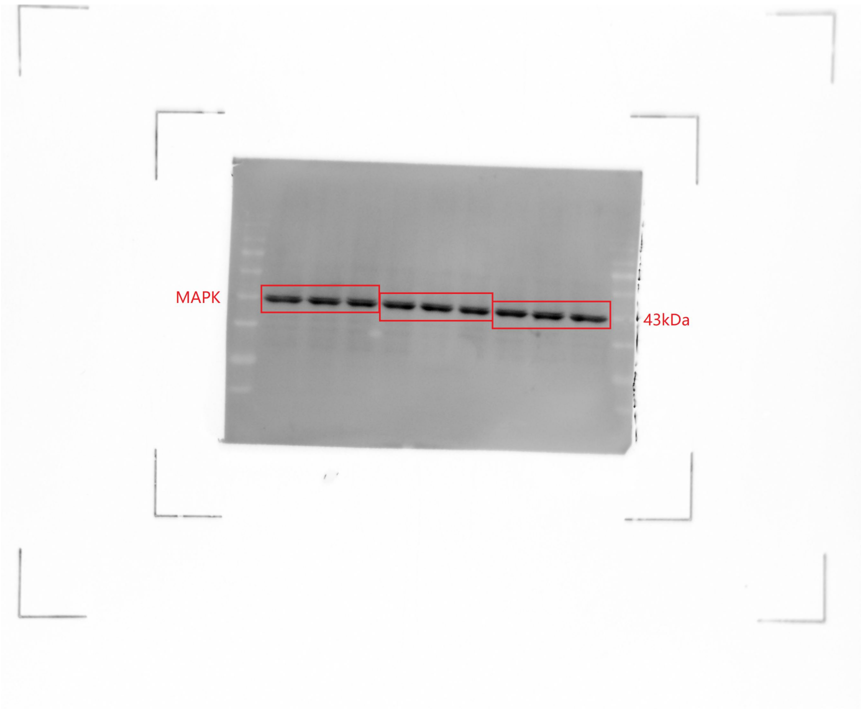

Figure 5A

The original blot of P-MAPK in K562 cells

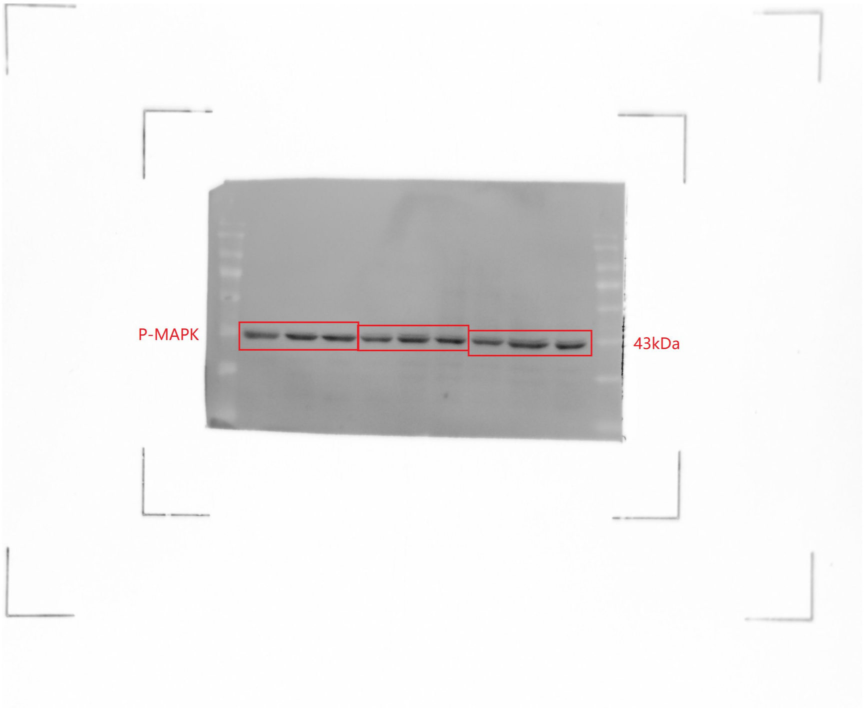

Figure 5B

The original blot of GAPDH in MEG-01 cells

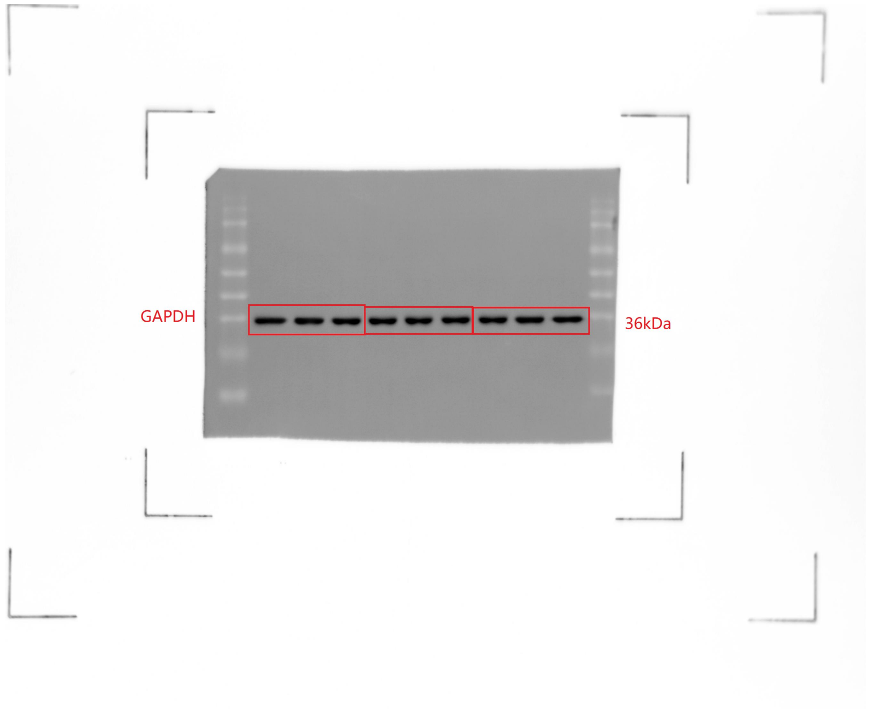

Figure 5B

The original blot of PI3K in MEG-01 cells

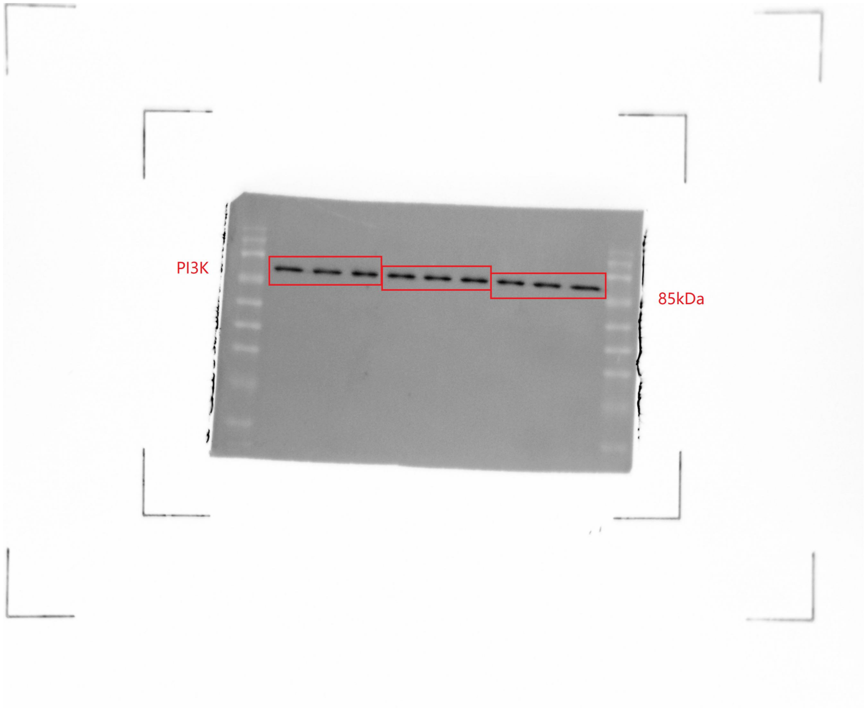

Figure 5B

The original blot of p-PI3K in MEG-01 cells

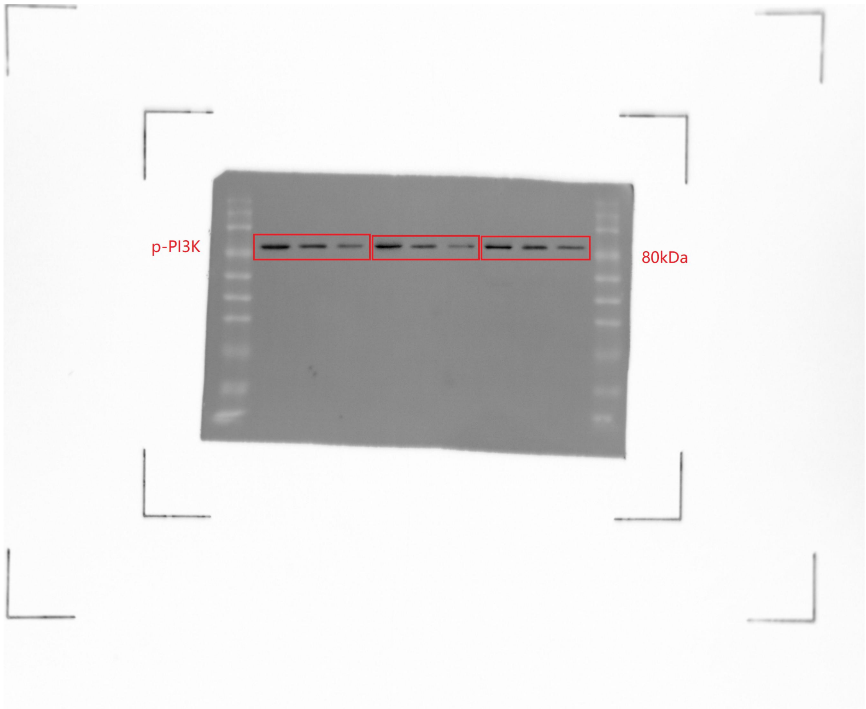

Figure 5B

The original blot of GAPDH in MEG-01 cells

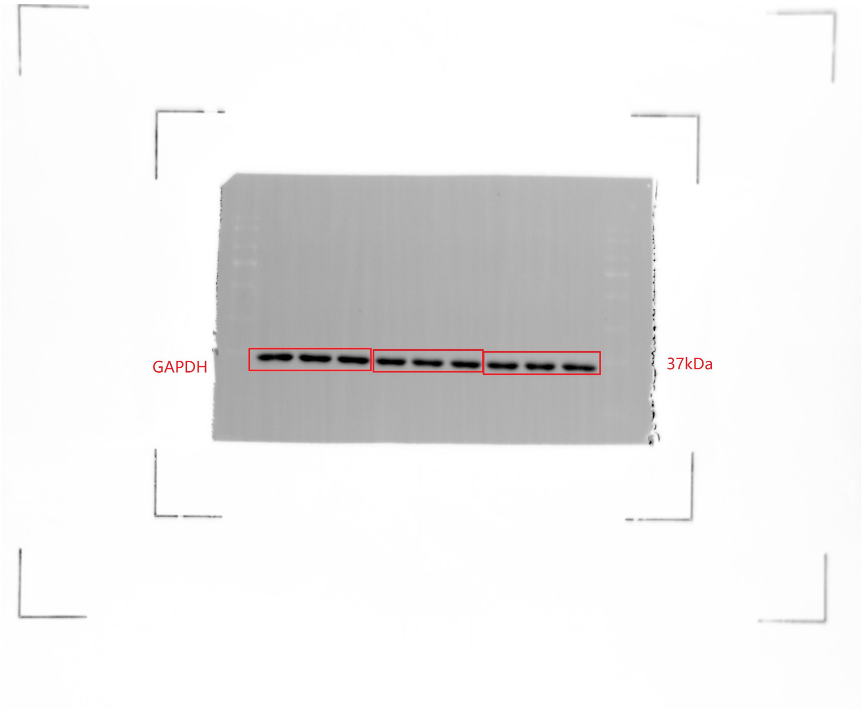

Figure 5B

The original blot of AKT in MEG-01 cells

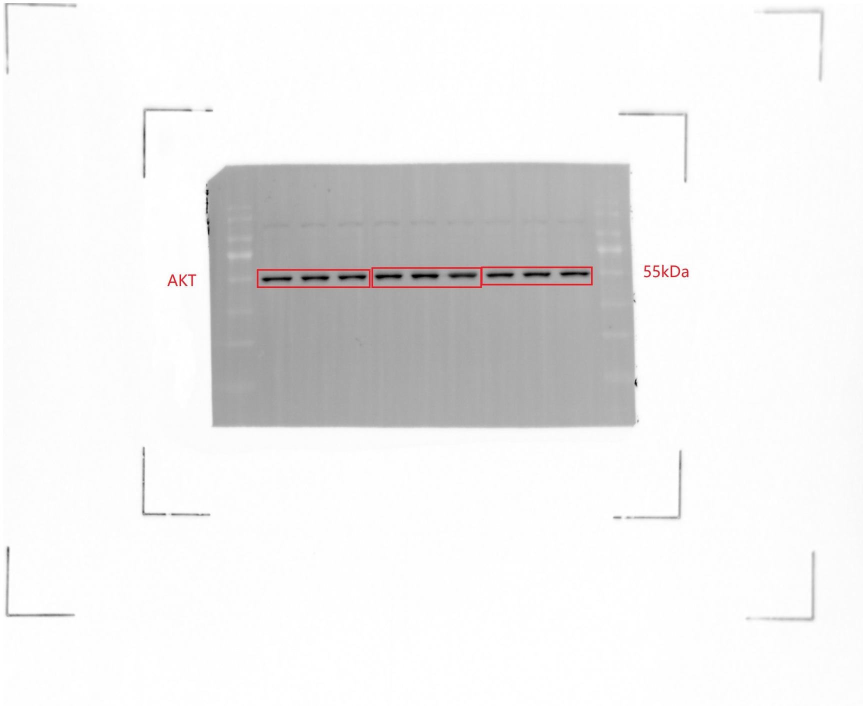

Figure 5B

The original blot of P-AKT in MEG-01 cells

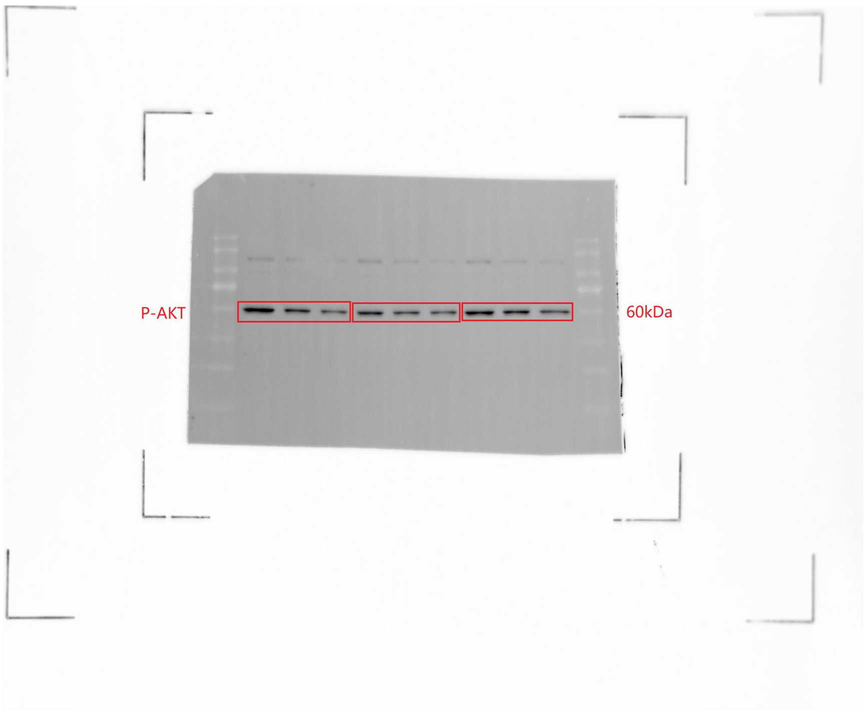

Figure 5B

The original blot of mTOR in MEG-01 cells

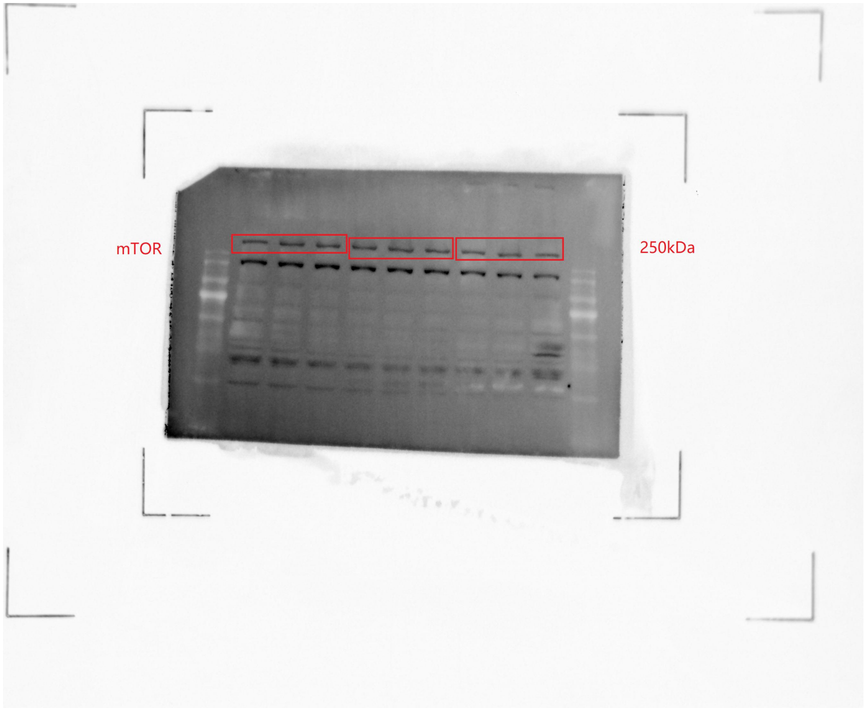

Figure 5B

The original blot of P-mTOR in MEG-01 cells

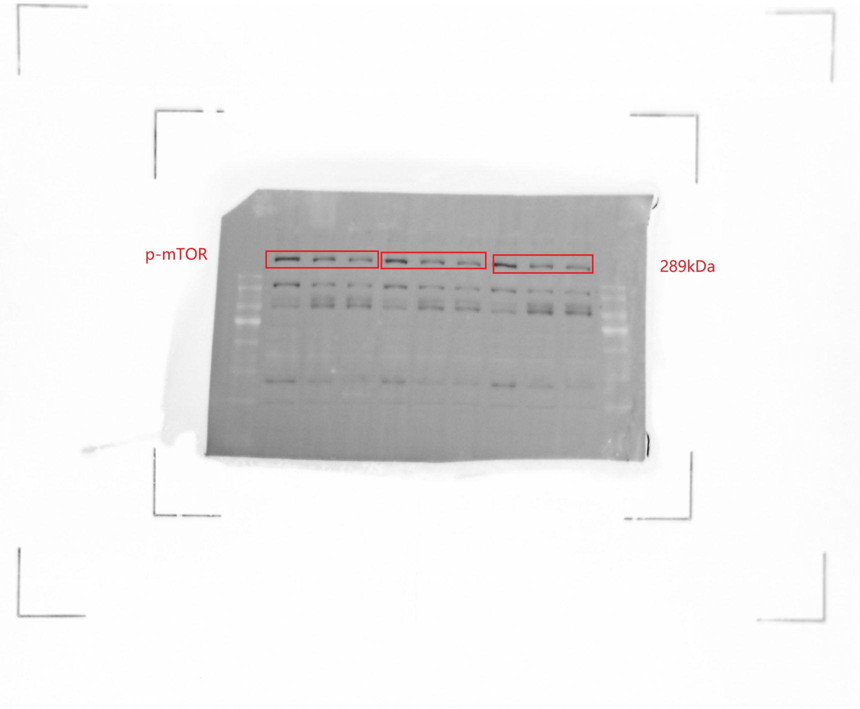

Figure 5B

The original blot of ERK in MEG-01 cells

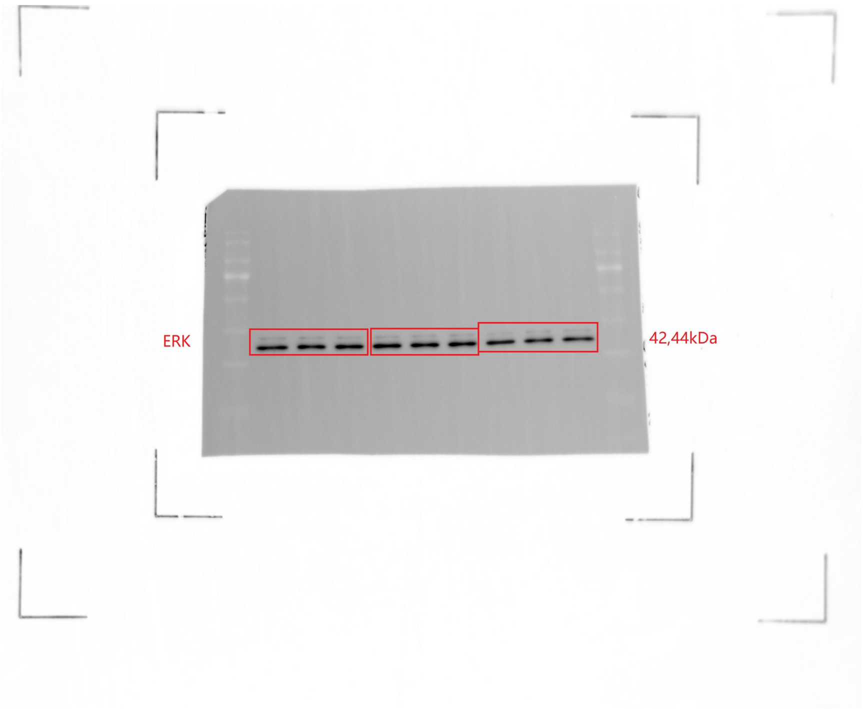

Figure 5B

The original blot of p-ERK in MEG-01 cells

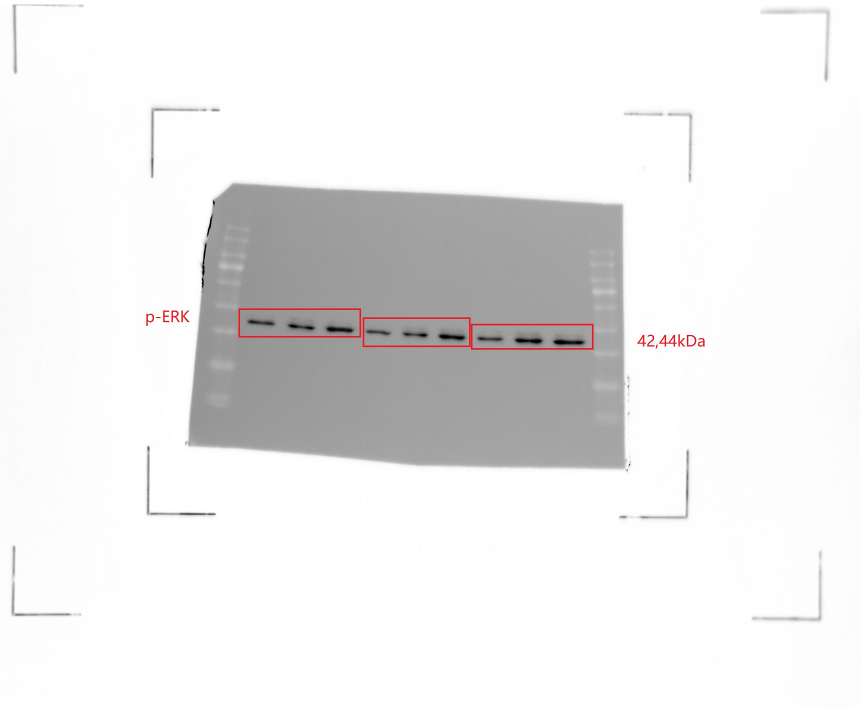

Figure 5B

The original blot of MAPK in MEG-01 cells

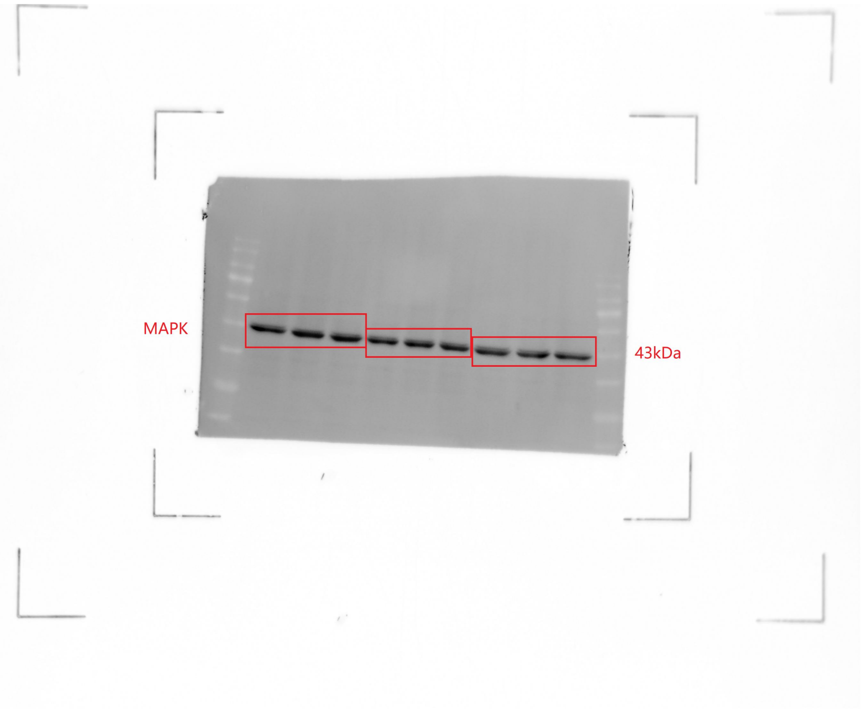

Figure 5B

The original blot of P-MAPK in MEG-01 cells

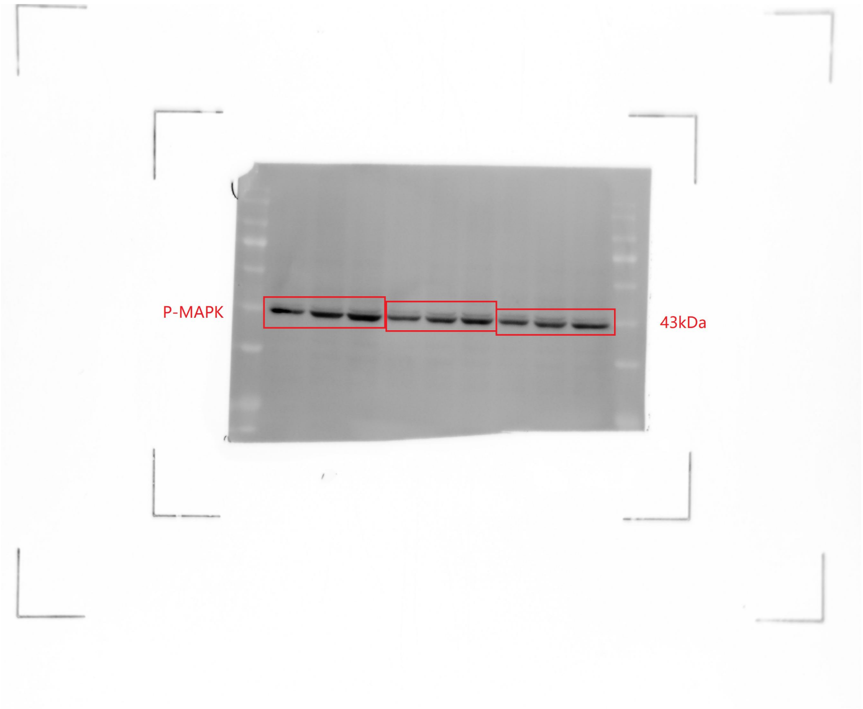

Figure 6C
The original blot of GAPDH in K562 cells

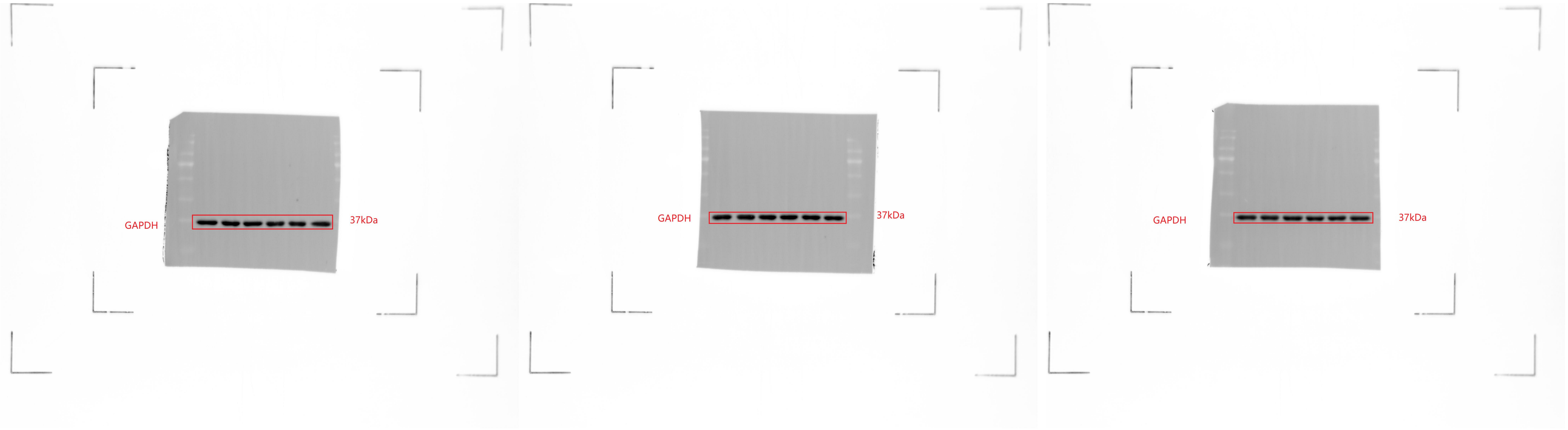

Figure 6C

The original blot of Bcl-XL in K562 cells

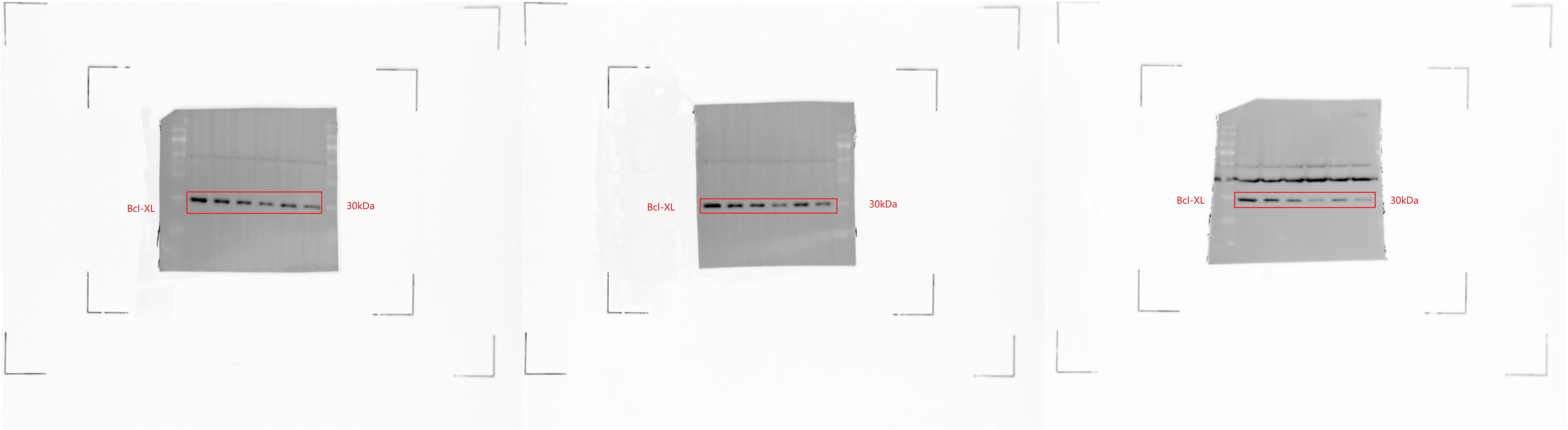

Figure 6C

The original blot of Bad in K562 cells

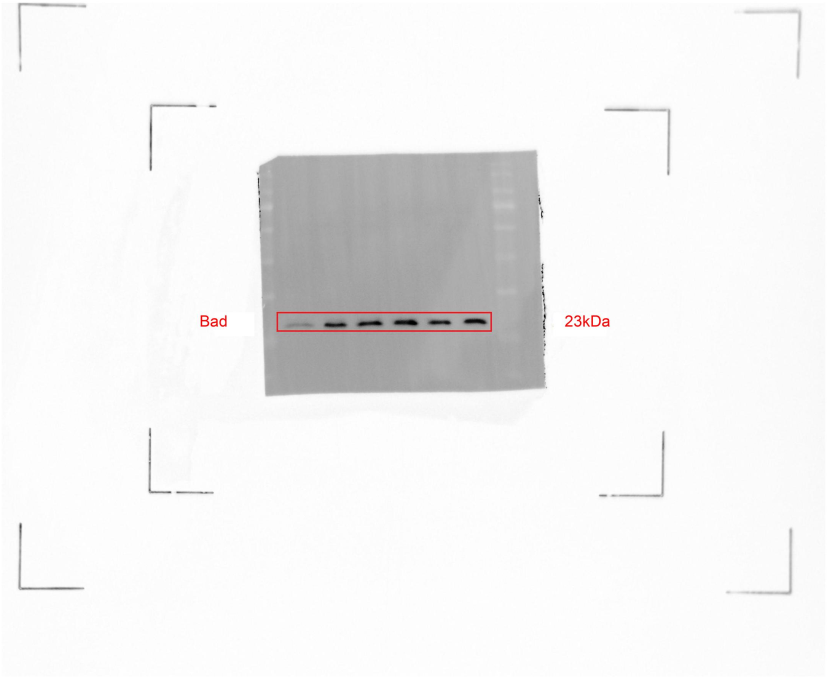

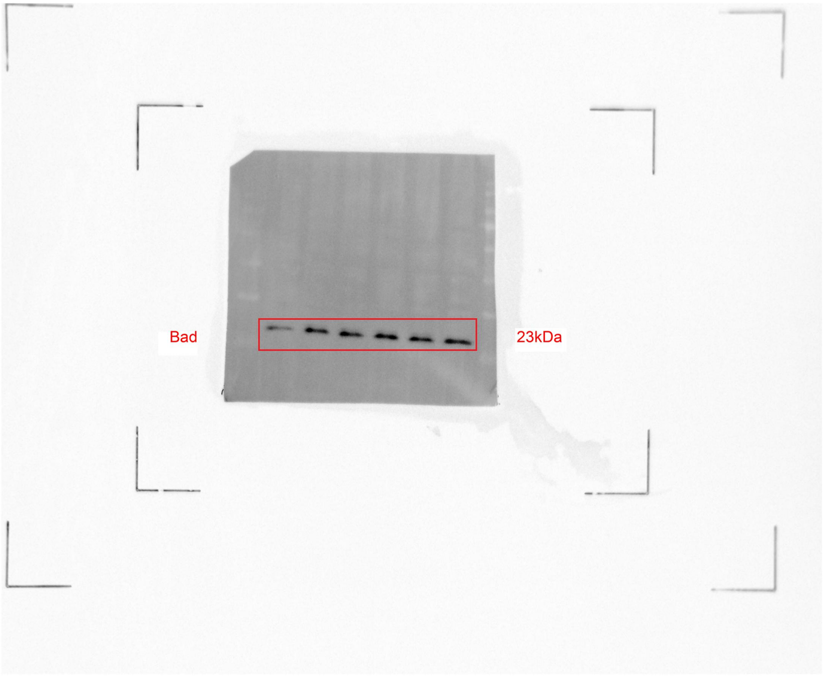

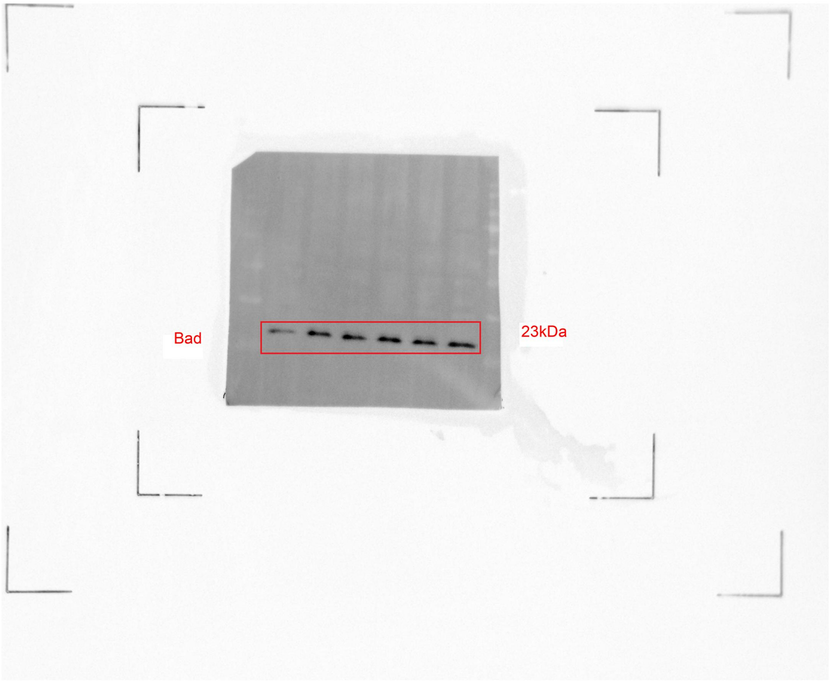

Figure 6C

The original blot of Survivin in K562 cells

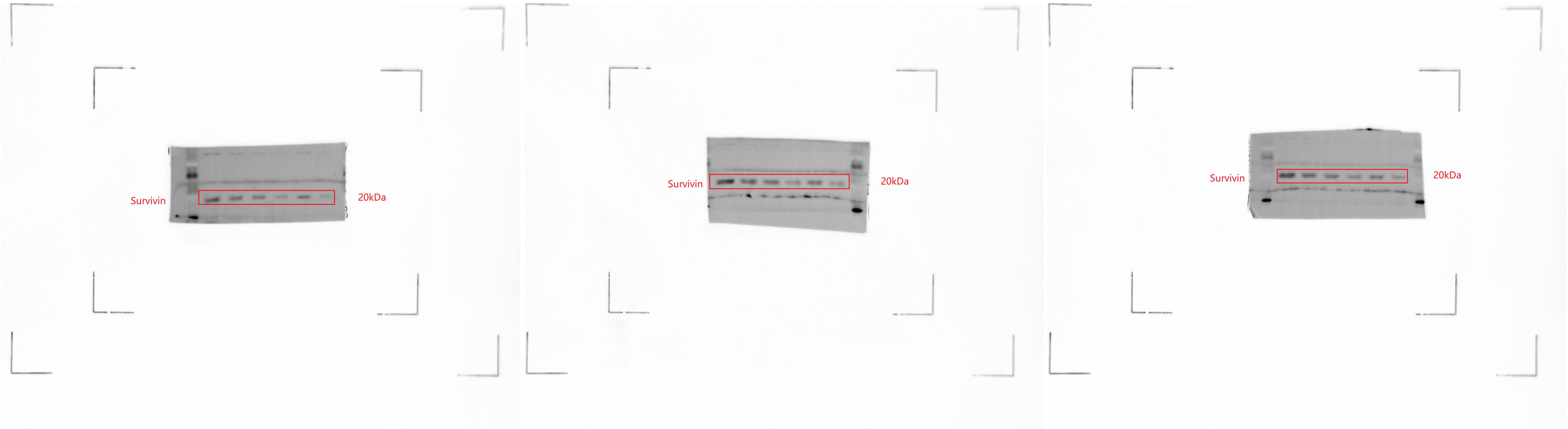

Figure 6C
The original blot of GAPDH in MEG-01 cells

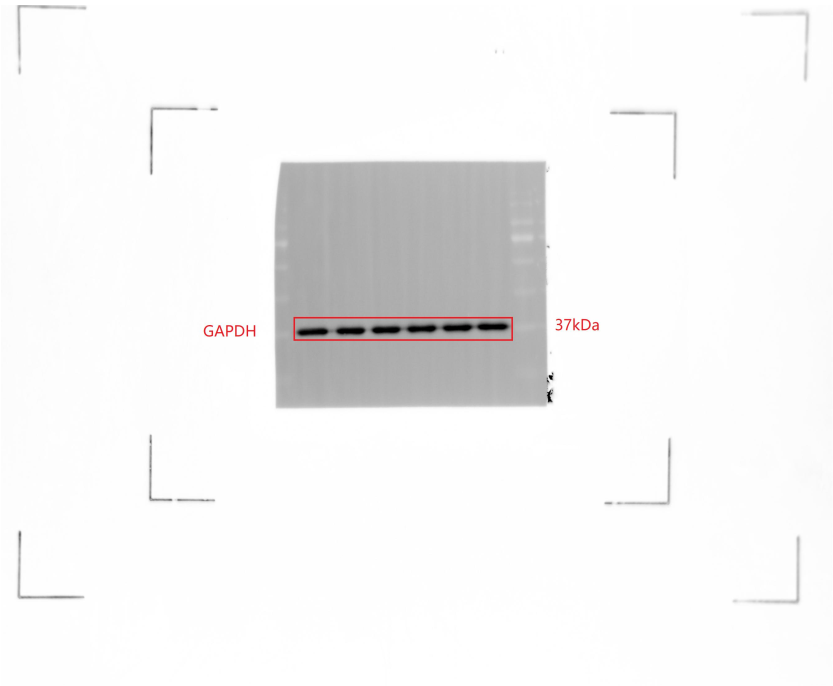

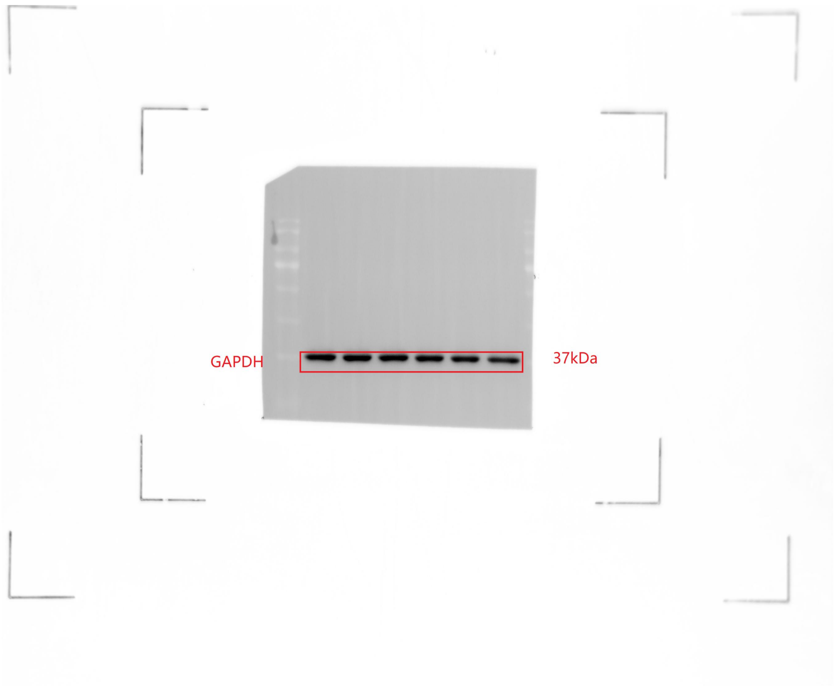

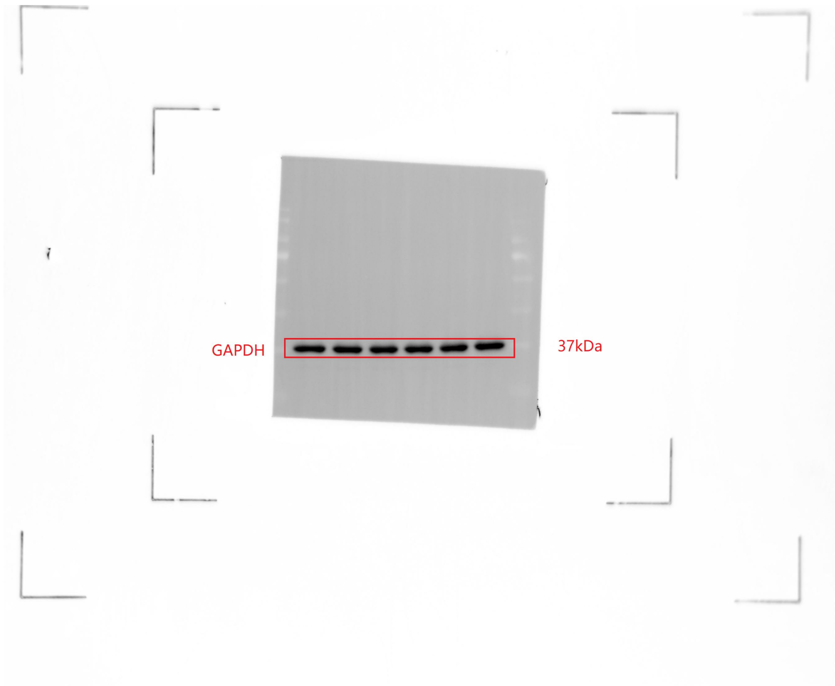

Figure 6C

The original blot of Bcl-XL in MEG-01 cells

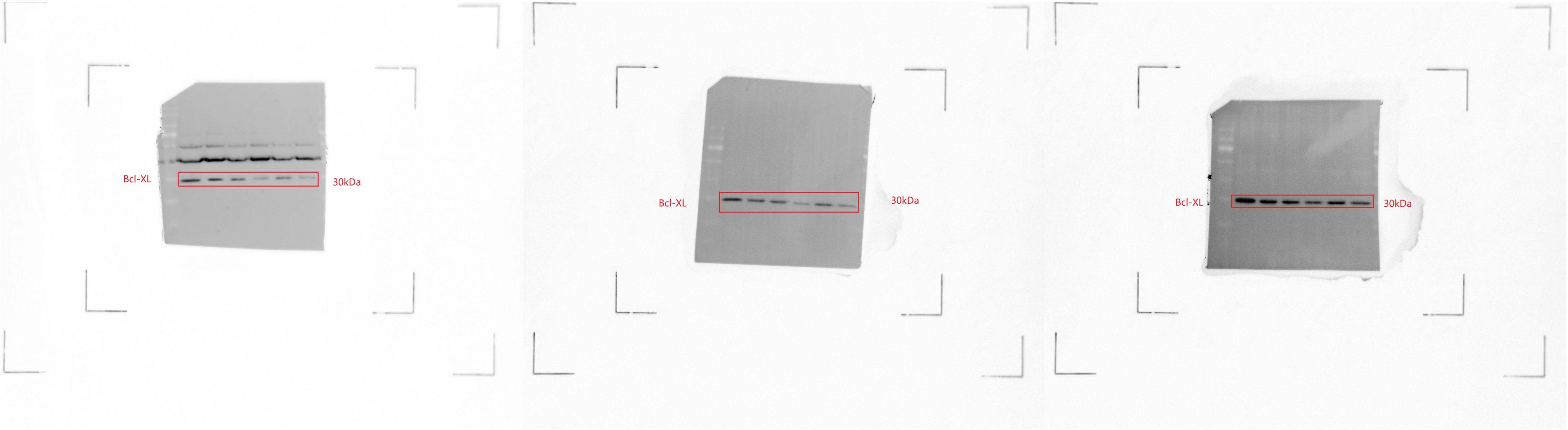

Figure 6C
The original blot of Bad in MEG-01 cells

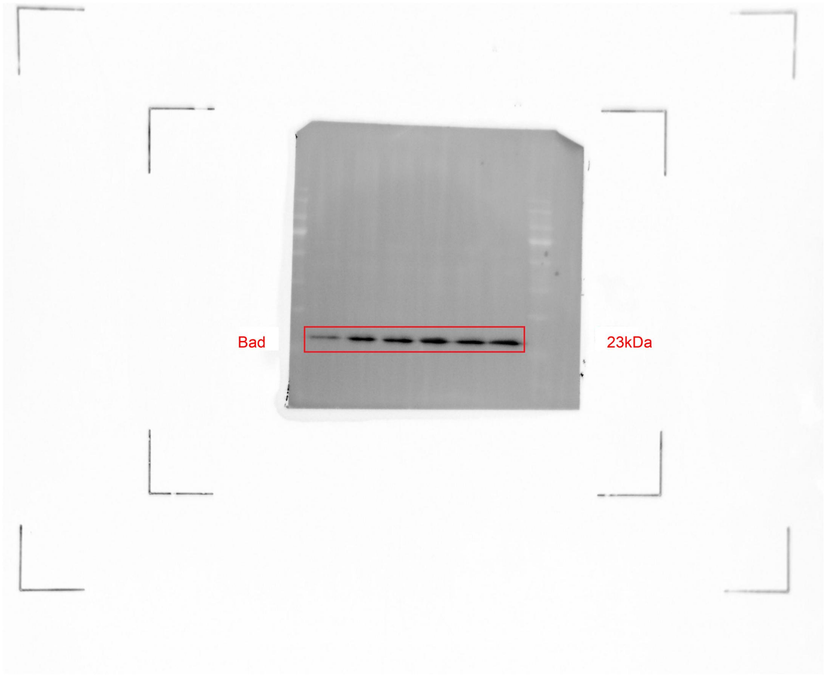

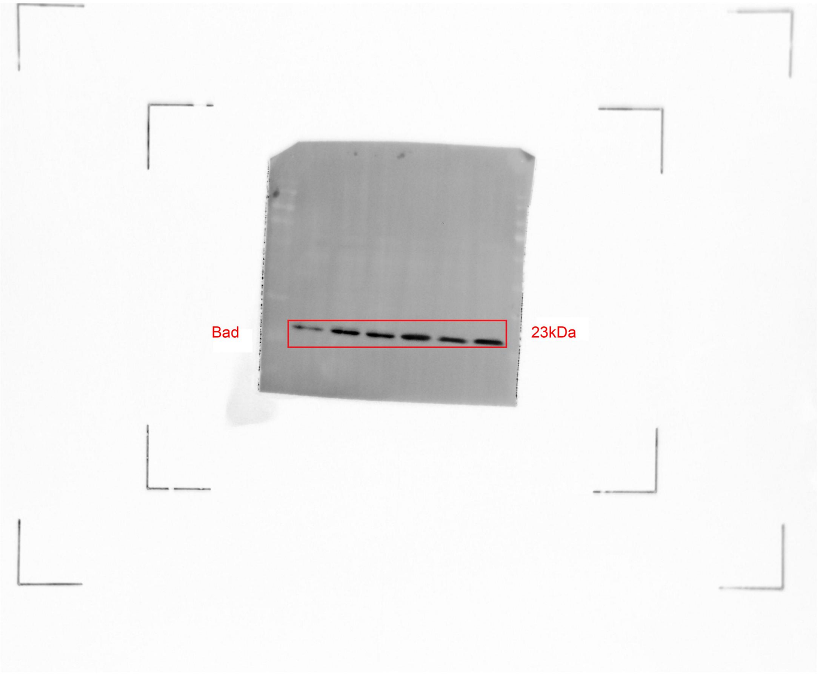

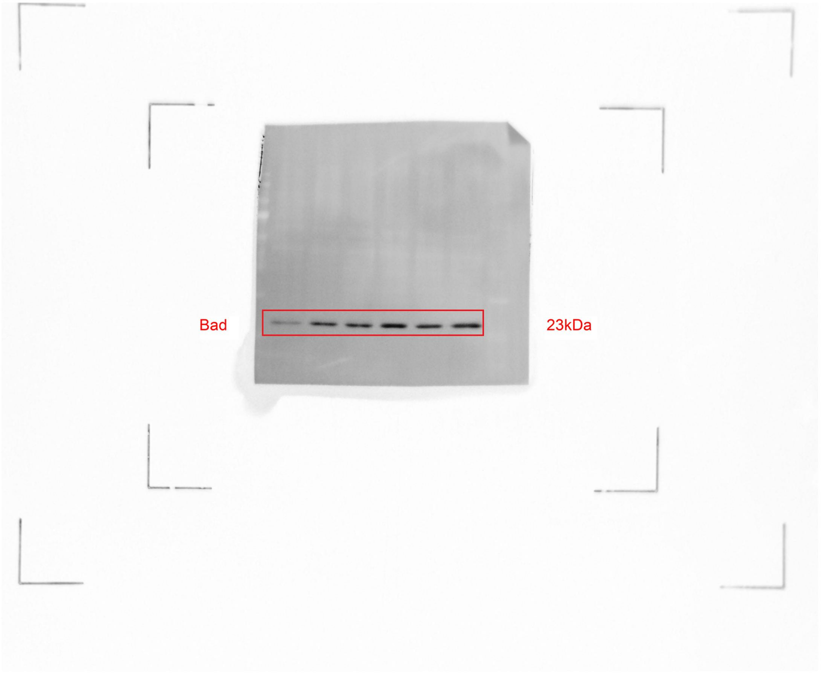

Figure 6C

The original blot of Survivin in MEG-01 cells

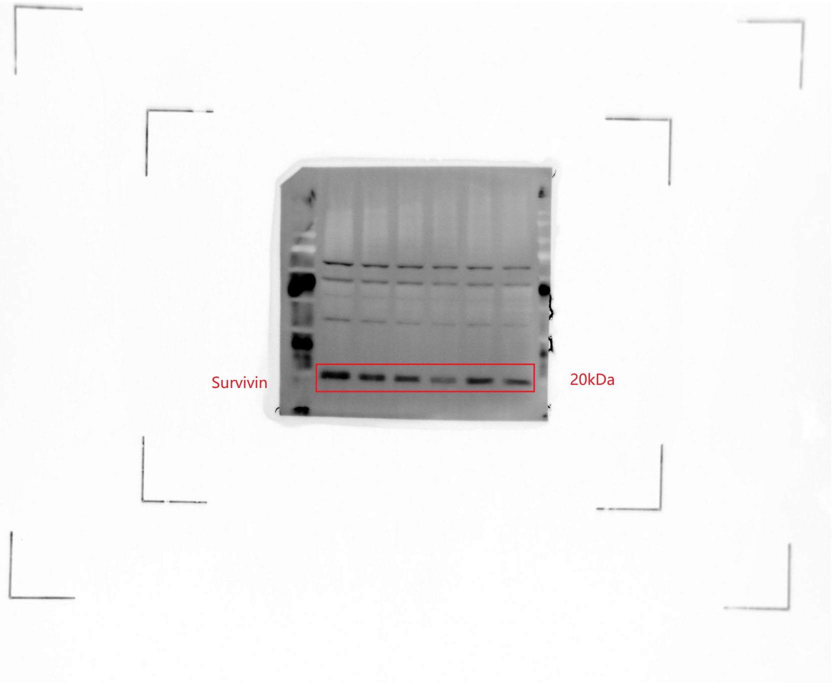

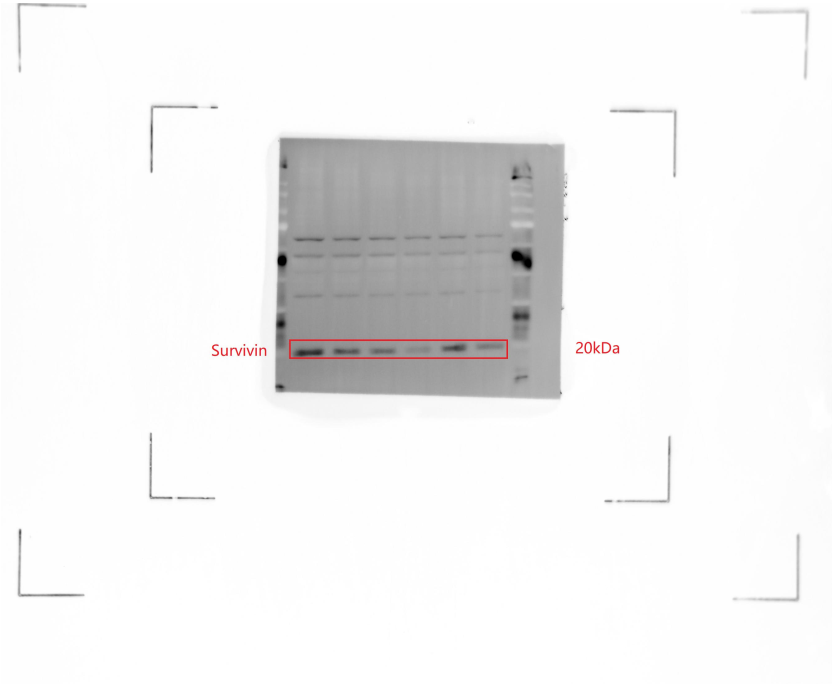

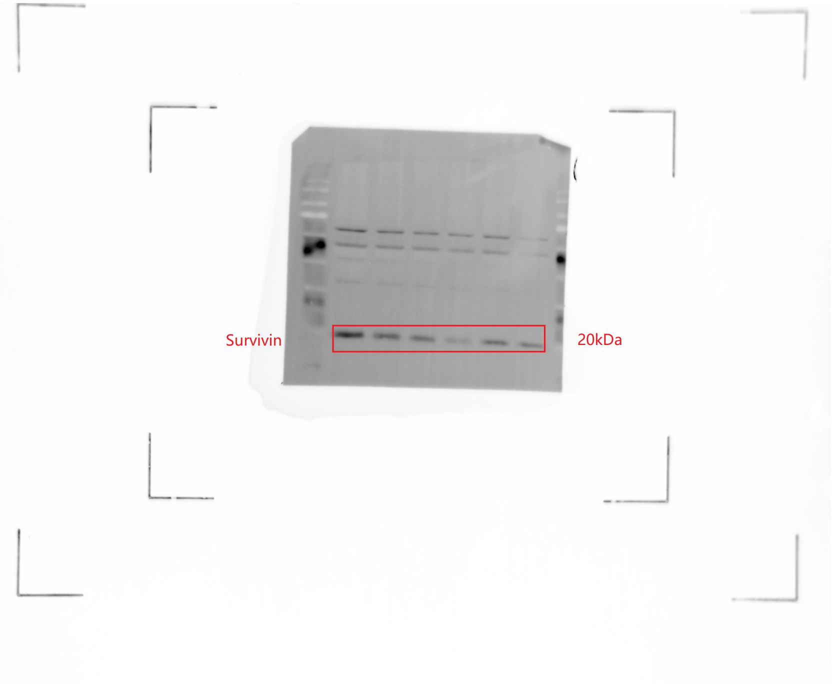

Figure 7B

The original blot of GAPDH in K562 cells

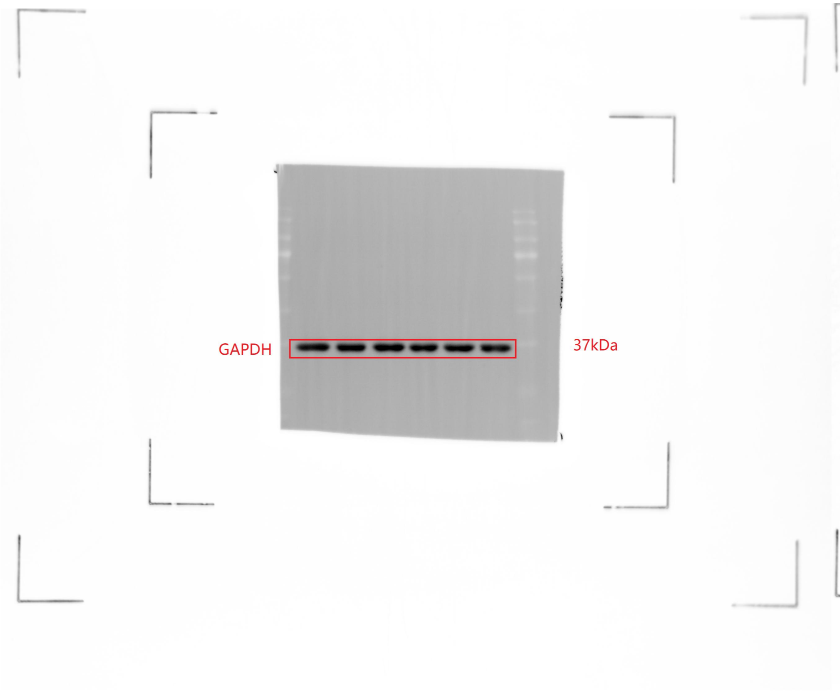

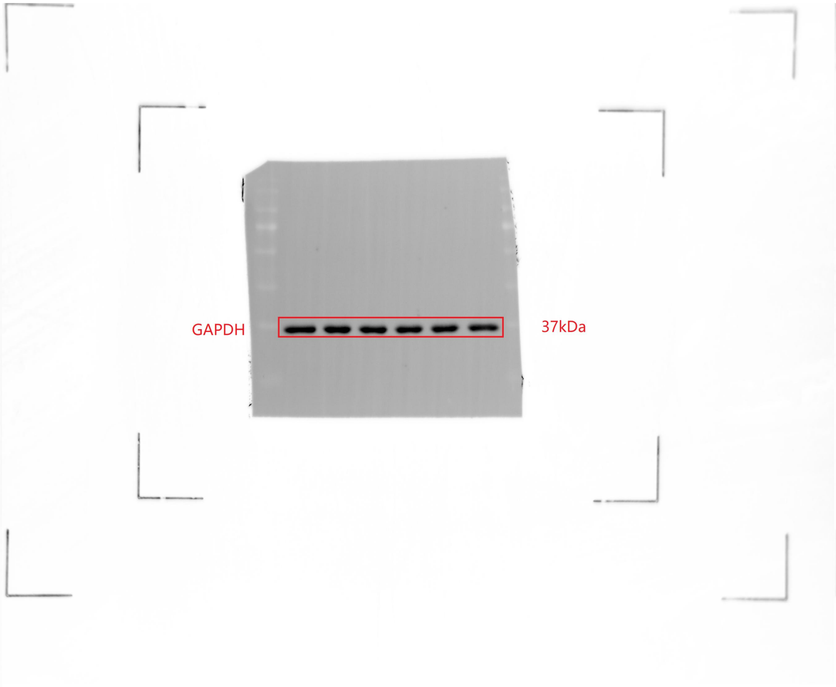

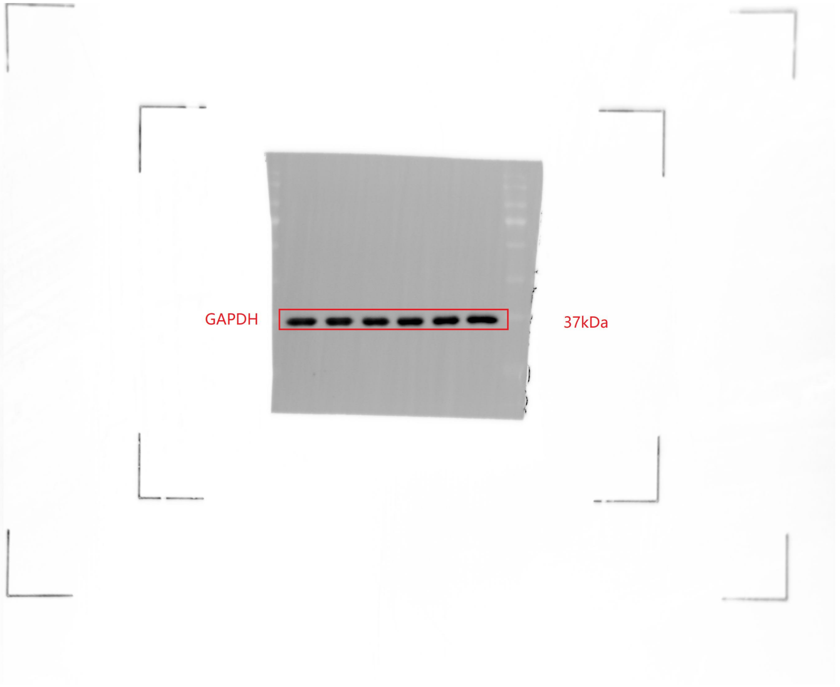

Figure 7B

The original blot of hTERT in K562 cells

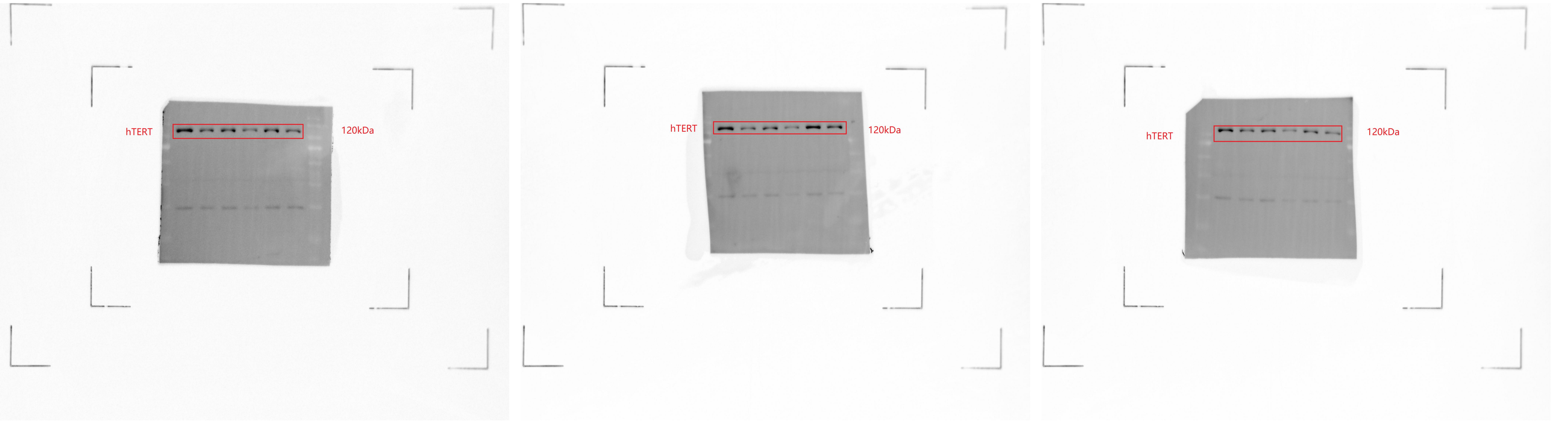

Figure 7B

The original blot of C-MYC in K562 cells

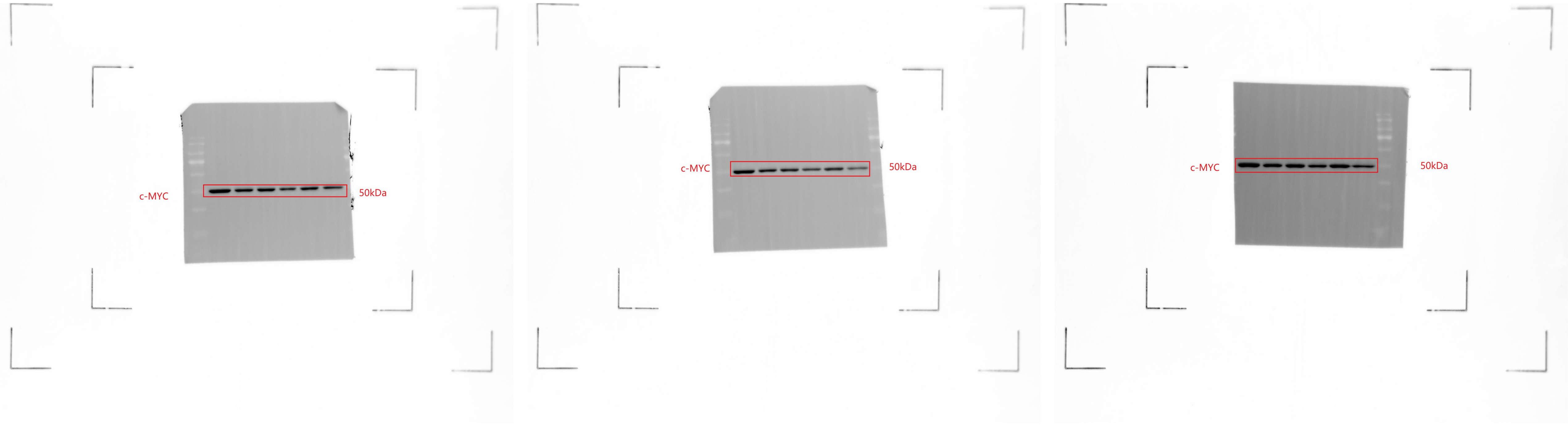

Figure 7B

The original blot of GAPDH in MEG-01 cells

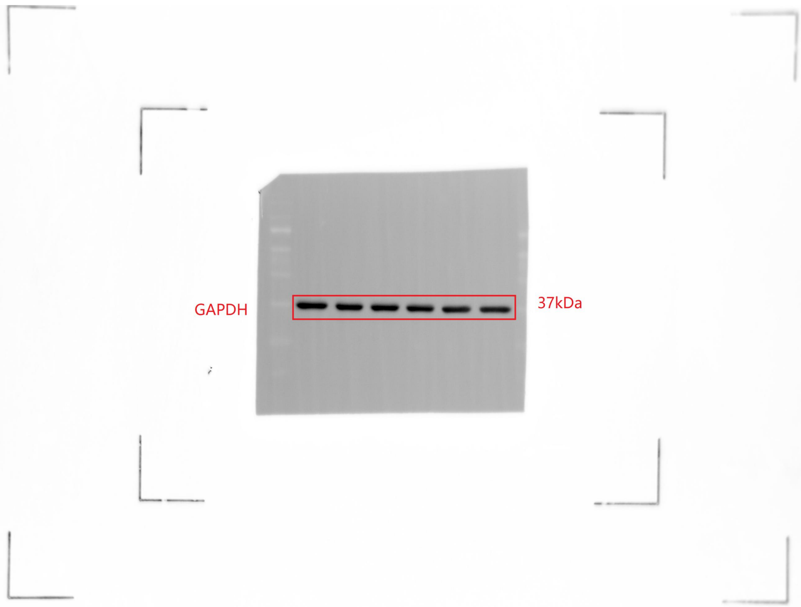

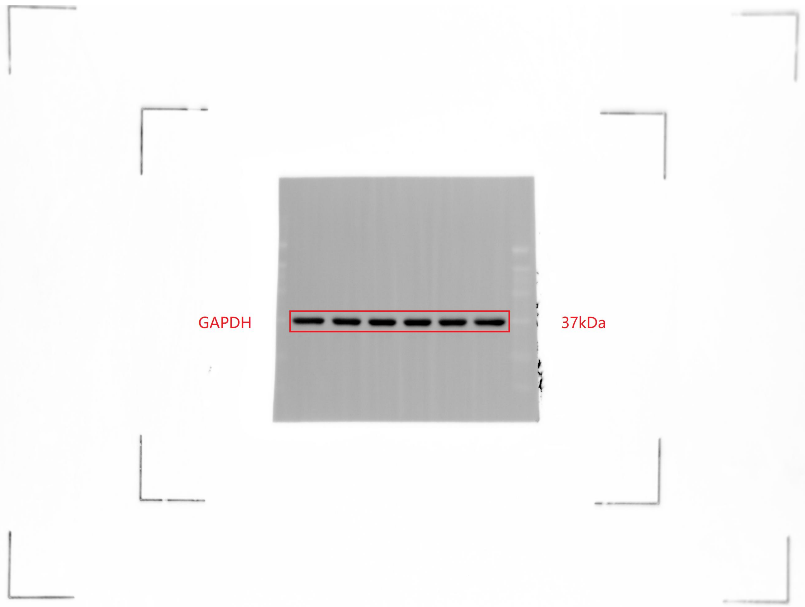

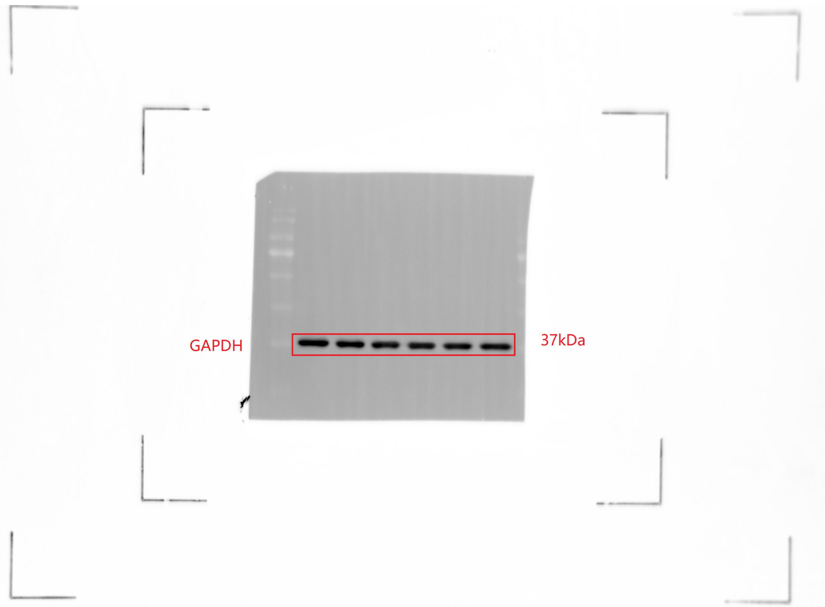

Figure 7B

The original blot of hTERT in MEG-01 cells

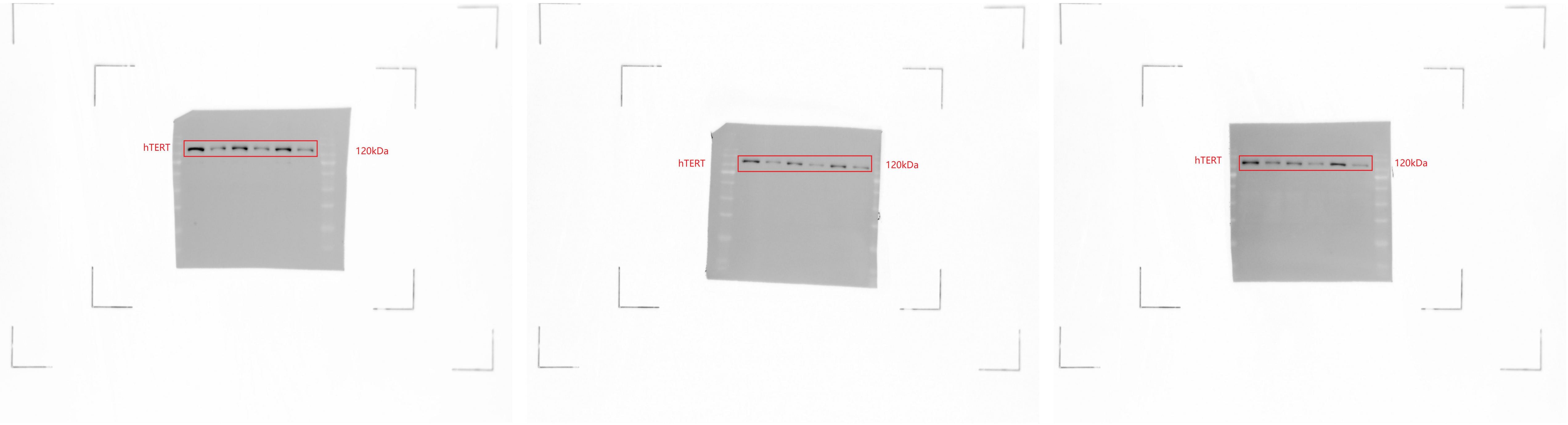

Figure 7B
The original blot of c-MYC in MEG-01 cells

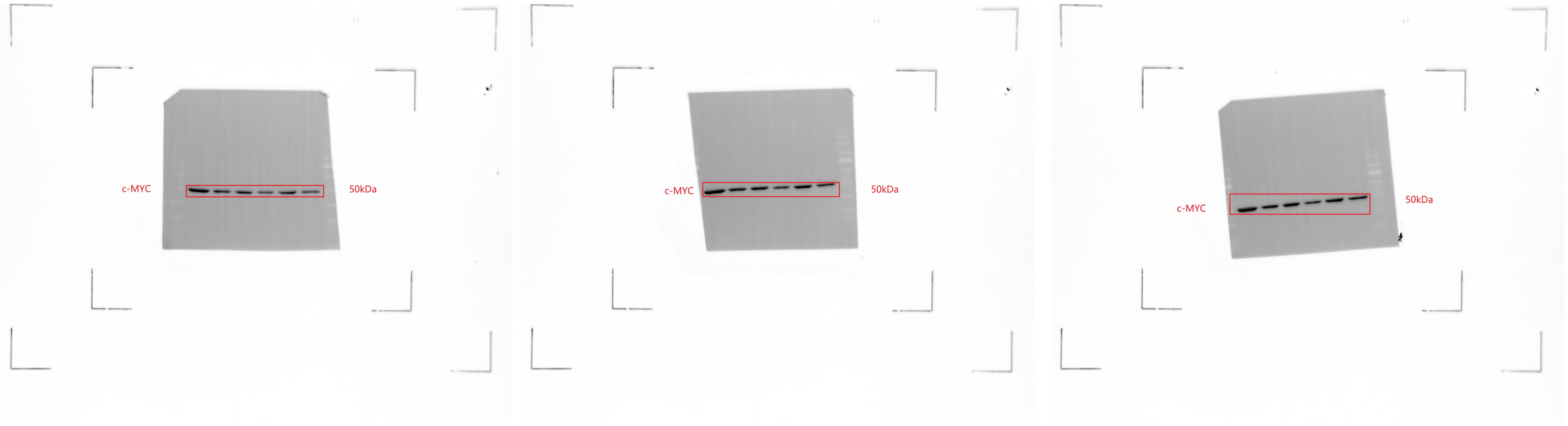

Figure 8A

The original blot of GAPDH in K562 cells

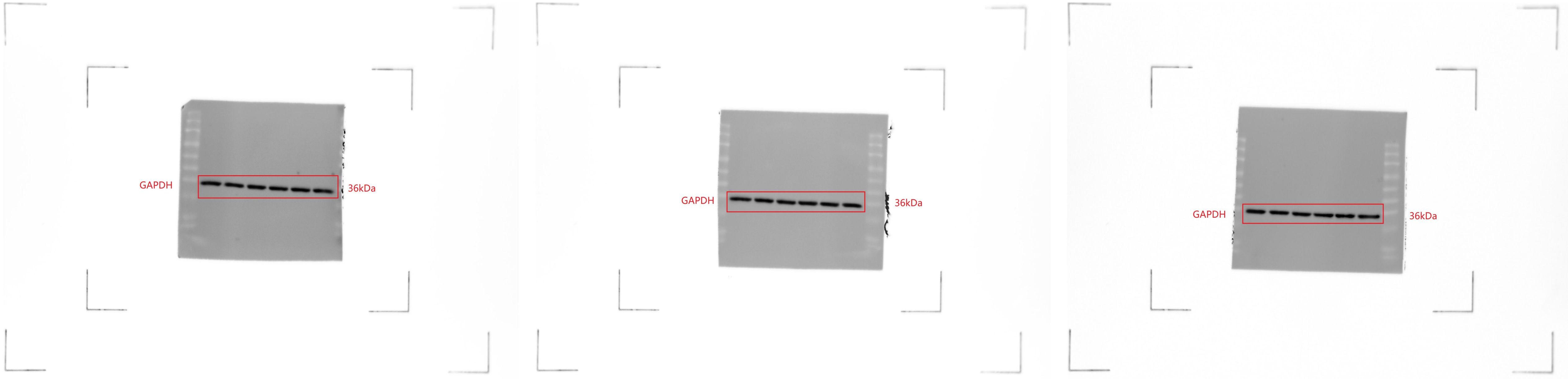

Figure 8A

The original blot of PI3K in K562 cells

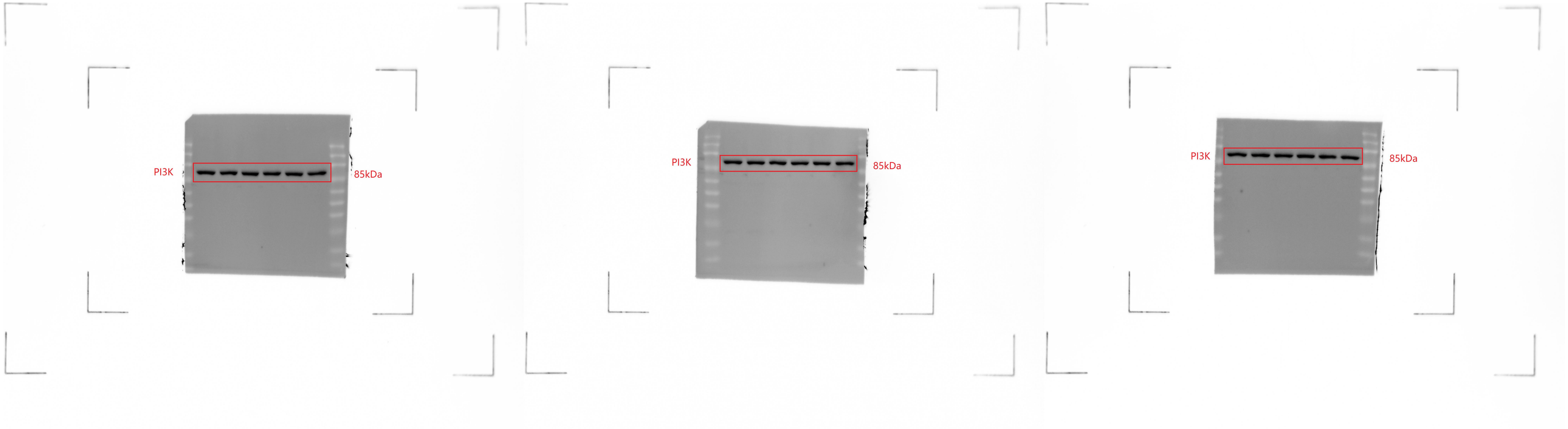

Figure 8A

The original blot of p-PI3K in K562 cells

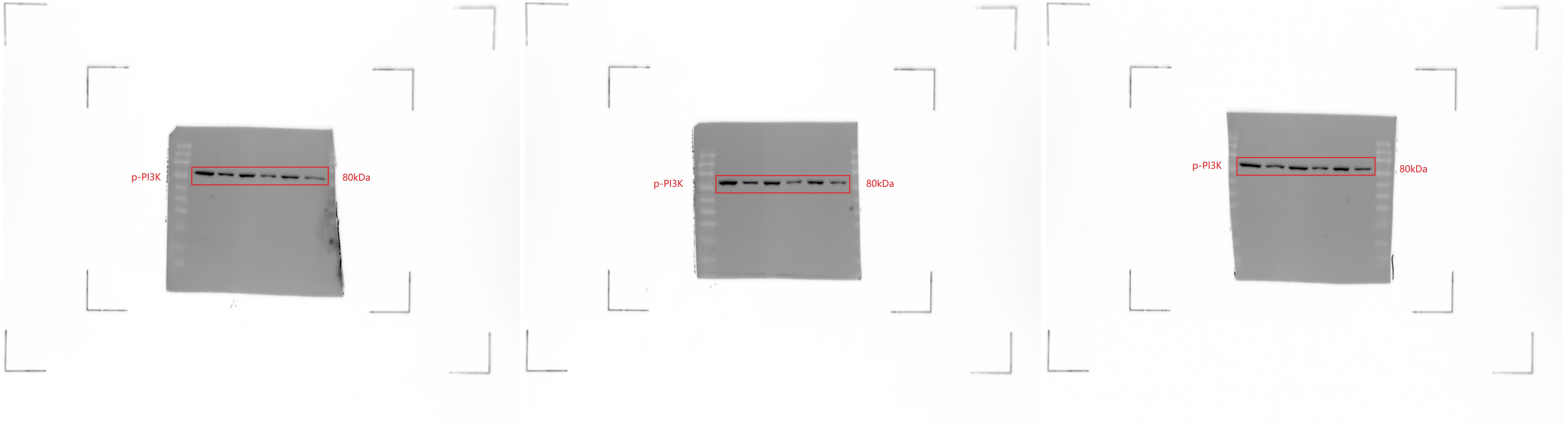

Figure 8A
The original blot of GAPDH in K562 cells

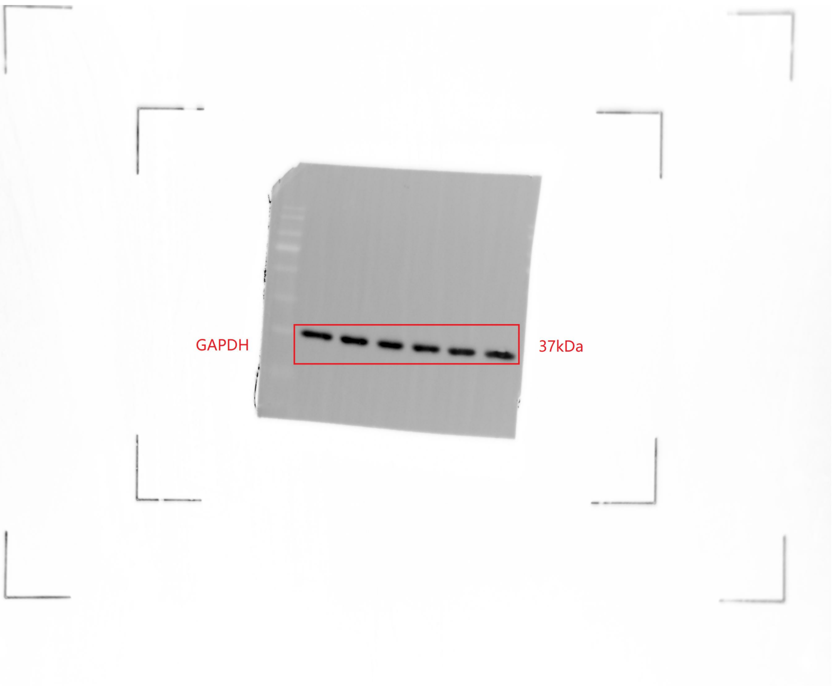

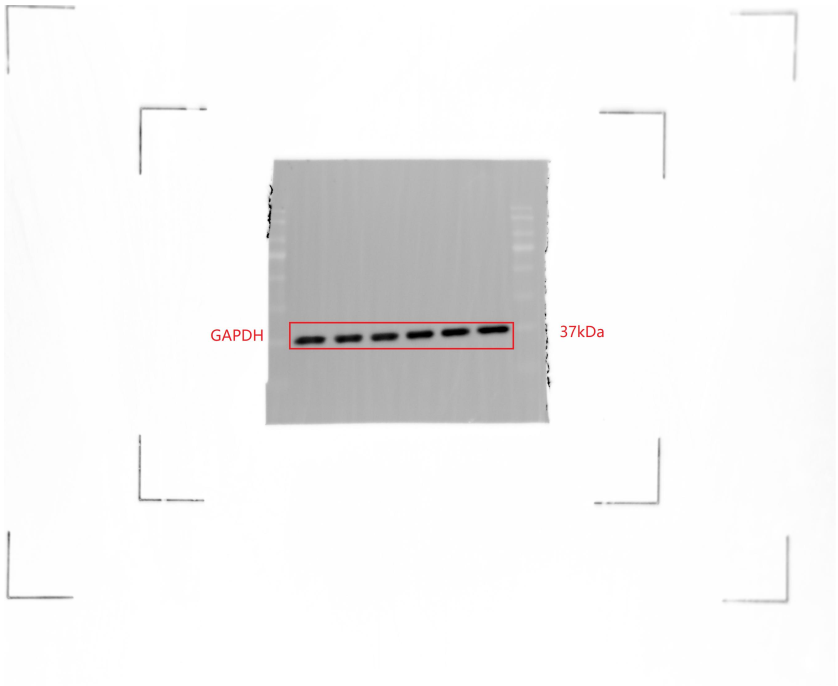

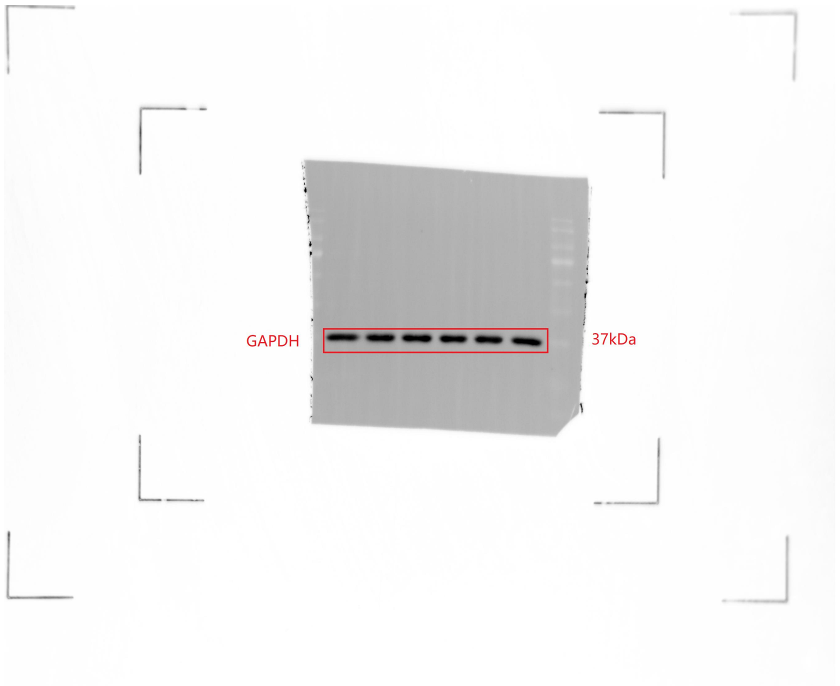

Figure 8A

The original blot of AKT in K562 cells

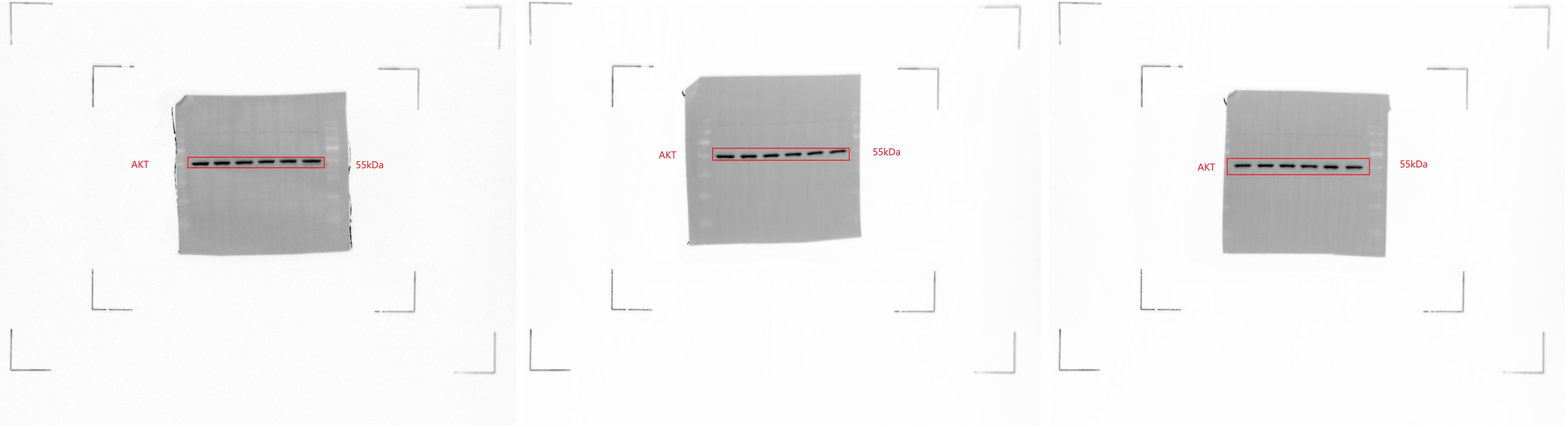

Figure 8A
The original blot of P-AKT in K562 cells

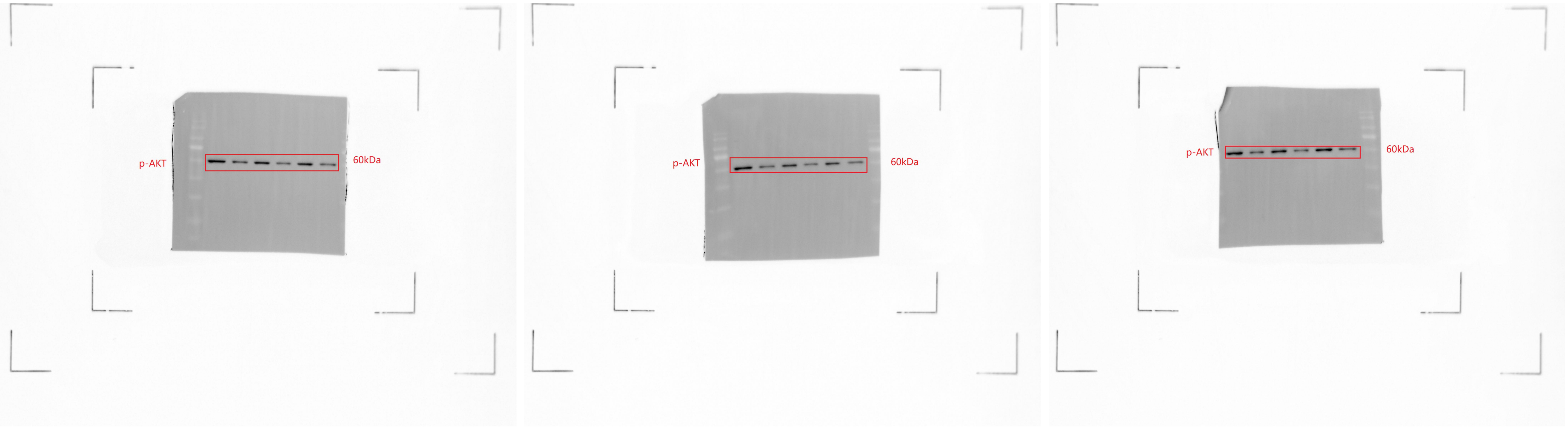

Figure 8A
The original blot of mTOR in K562 cells

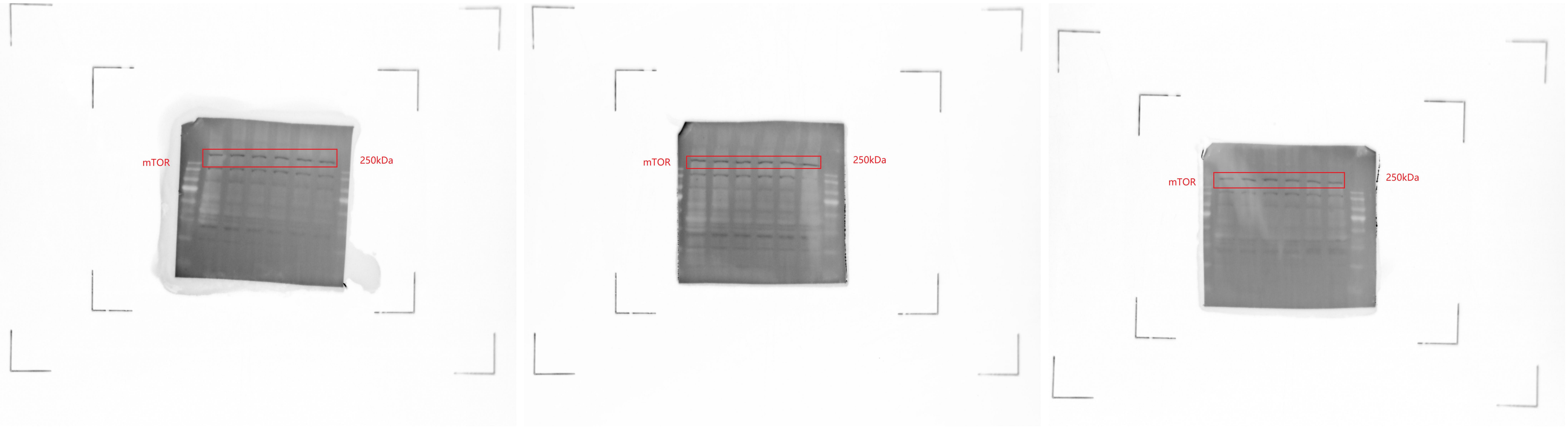

Figure 8A

The original blot of P-mTOR in K562 cells

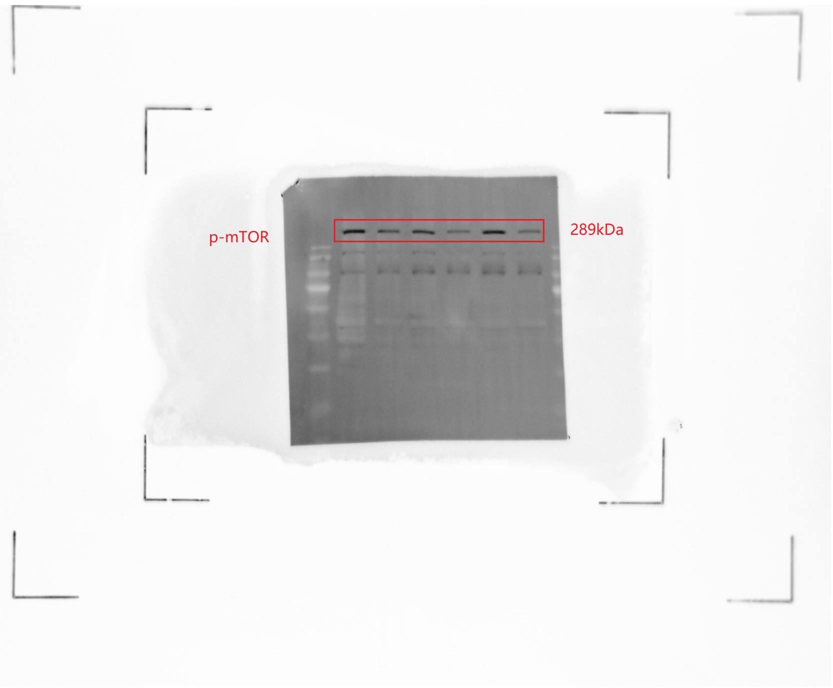

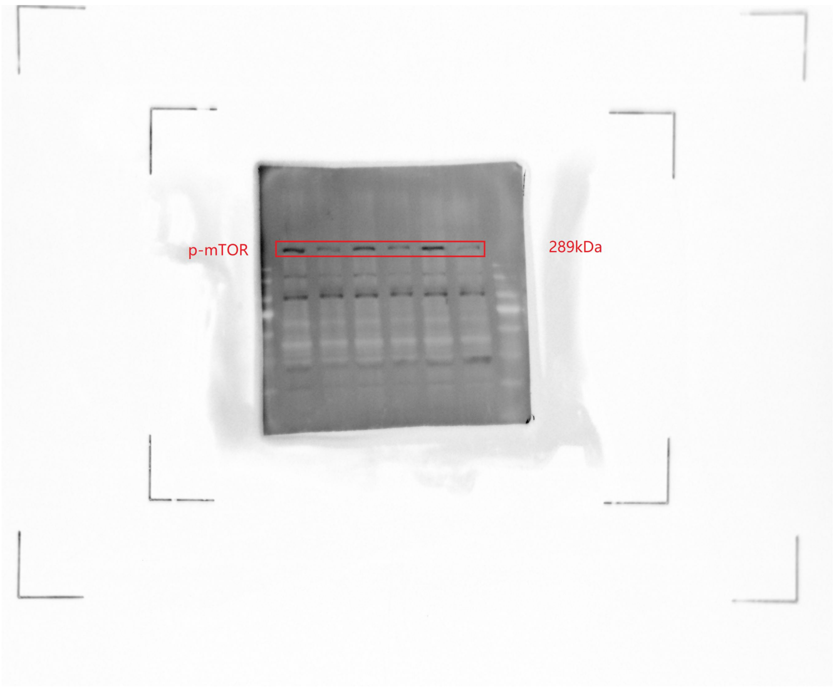

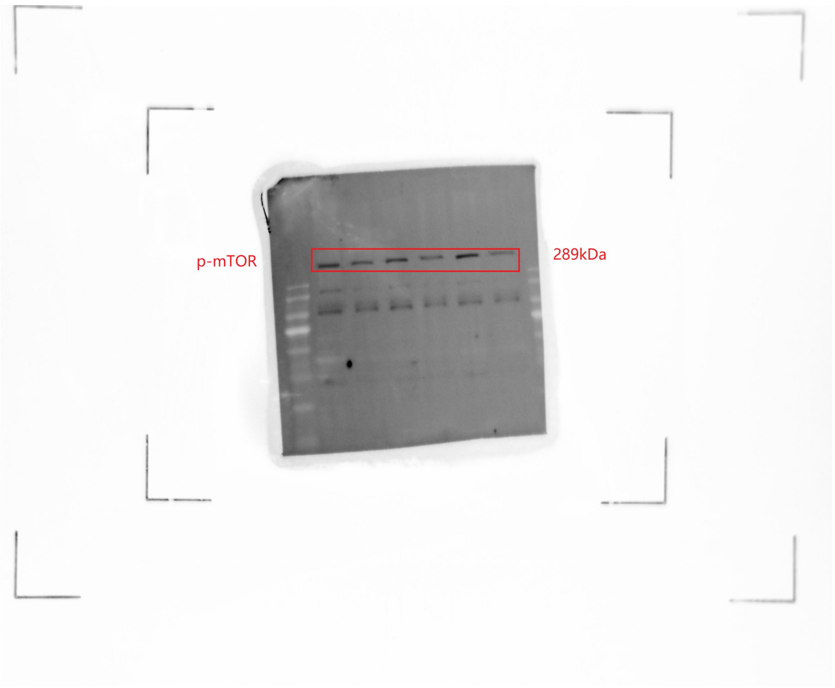

Figure 8A
The original blot of ERK in K562 cells

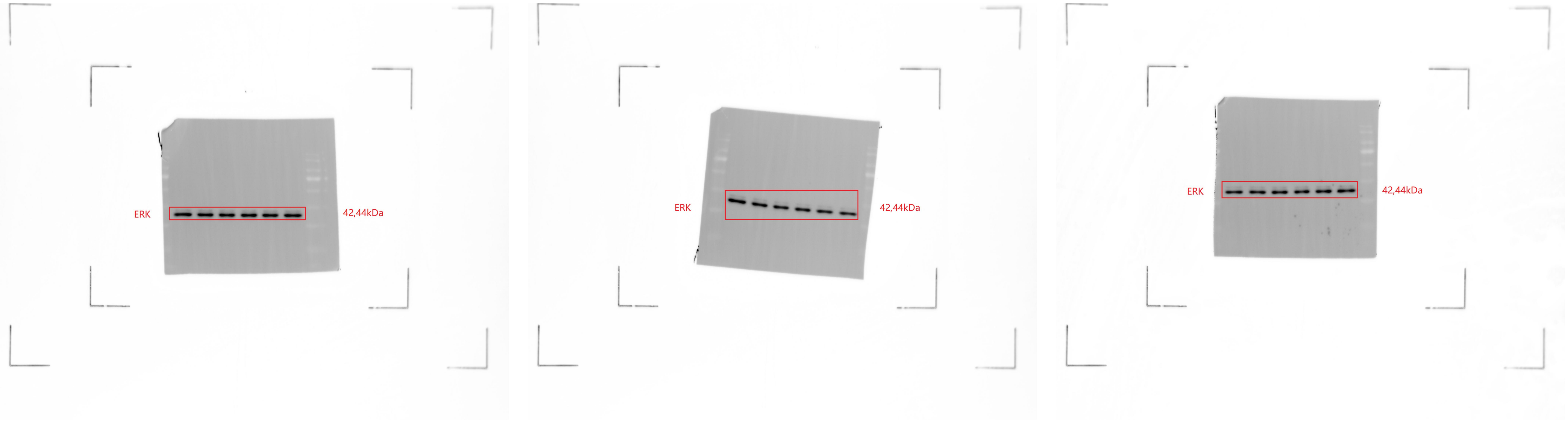

Figure 8A

The original blot of p-ERK in K562 cells

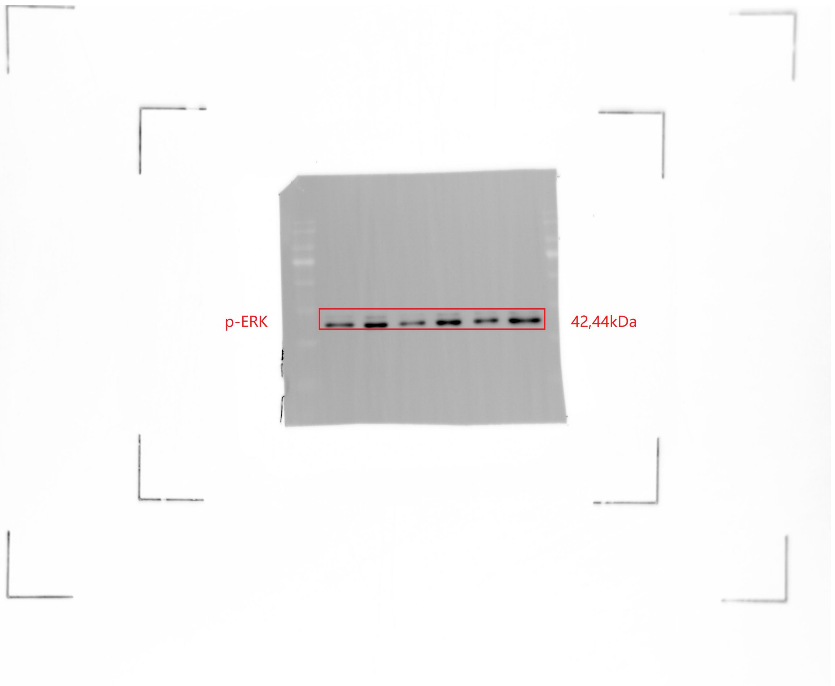

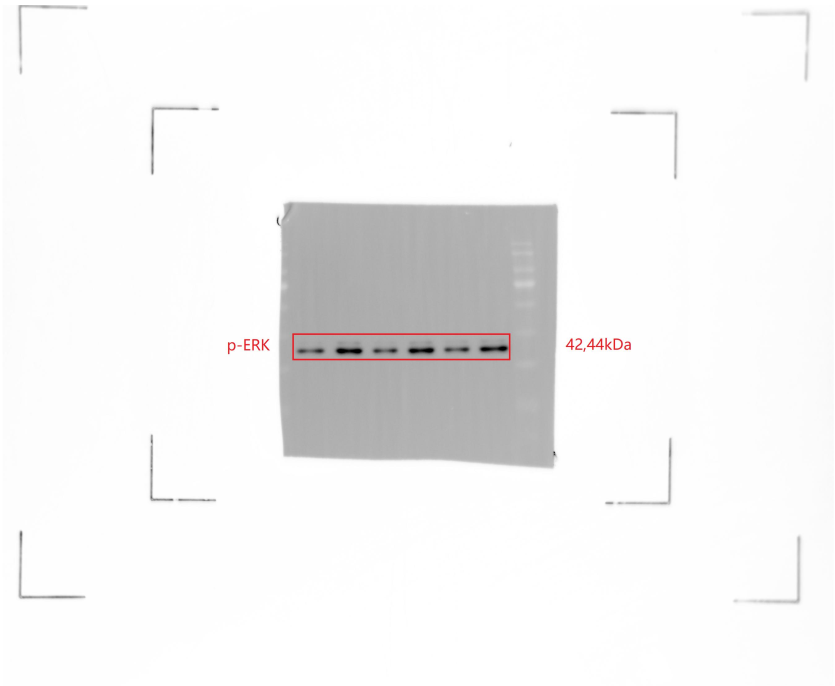

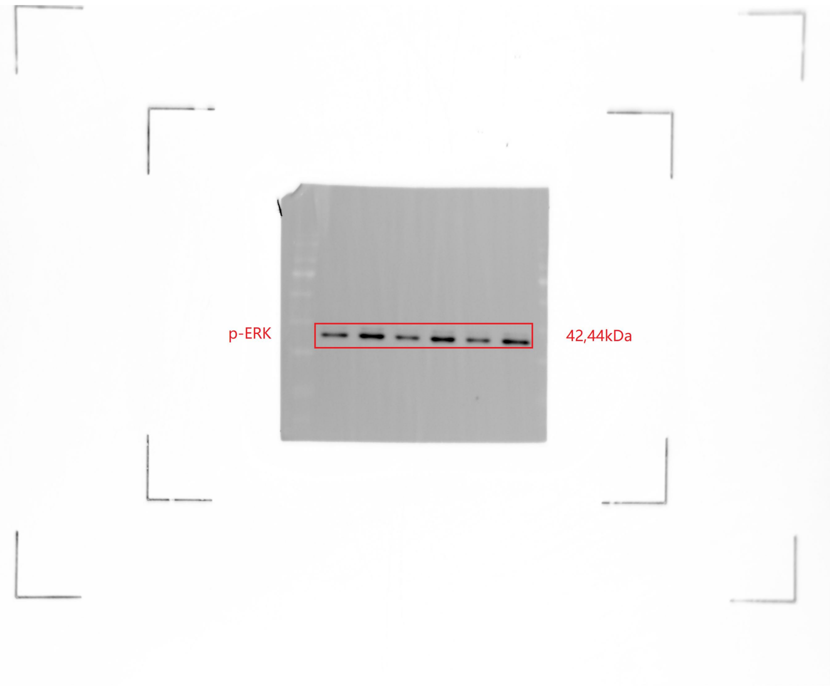

Figure 8A

The original blot of MAPK in K562 cells

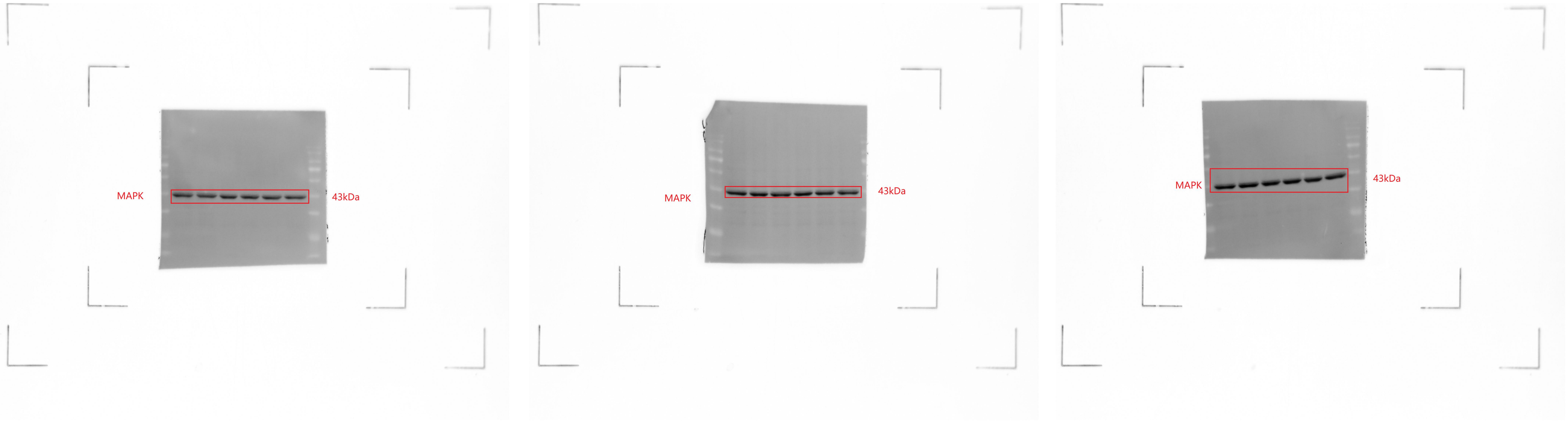

Figure 8A
The original blot of P-MAPK in K562 cells

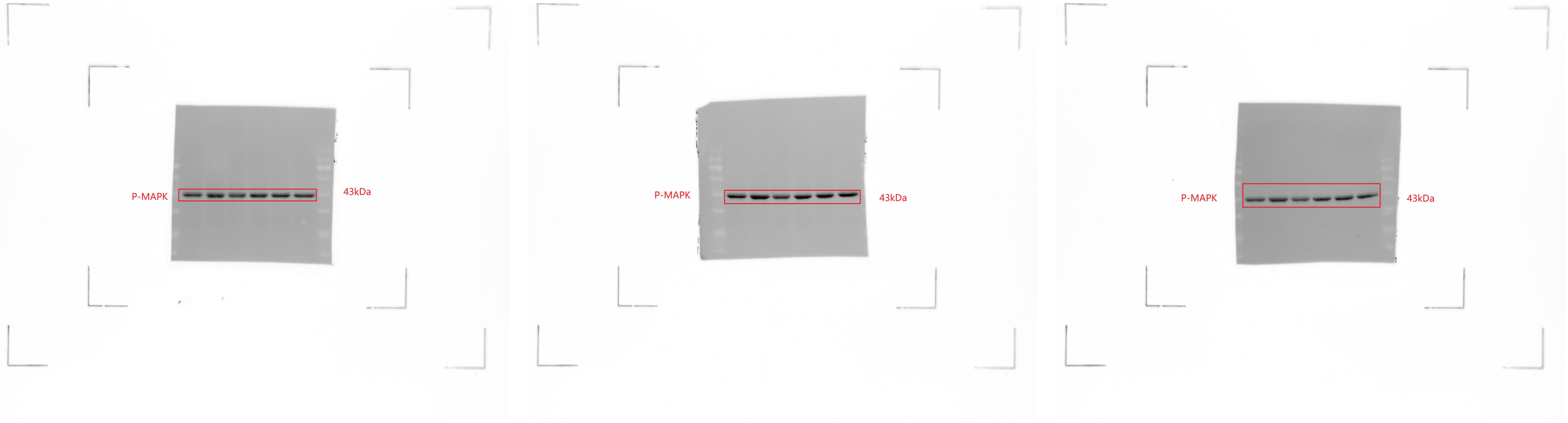

Figure 8B
The original blot of GAPDH in MEG-01 cells

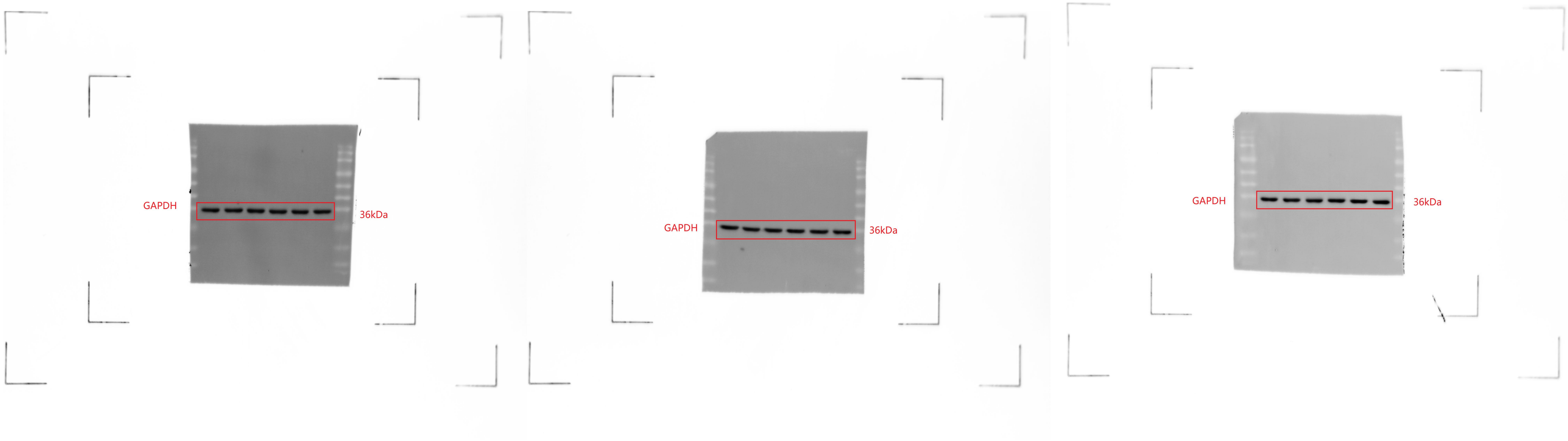

Figure 8B

The original blot of PI3K in MEG-01 cells

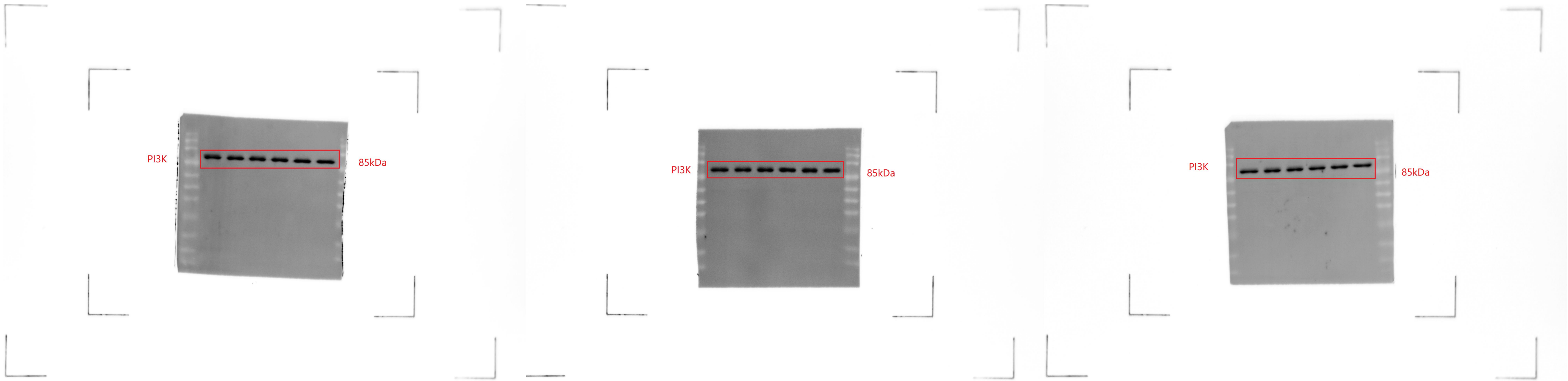

Figure 8B

The original blot of p-PI3K in MEG-01 cells

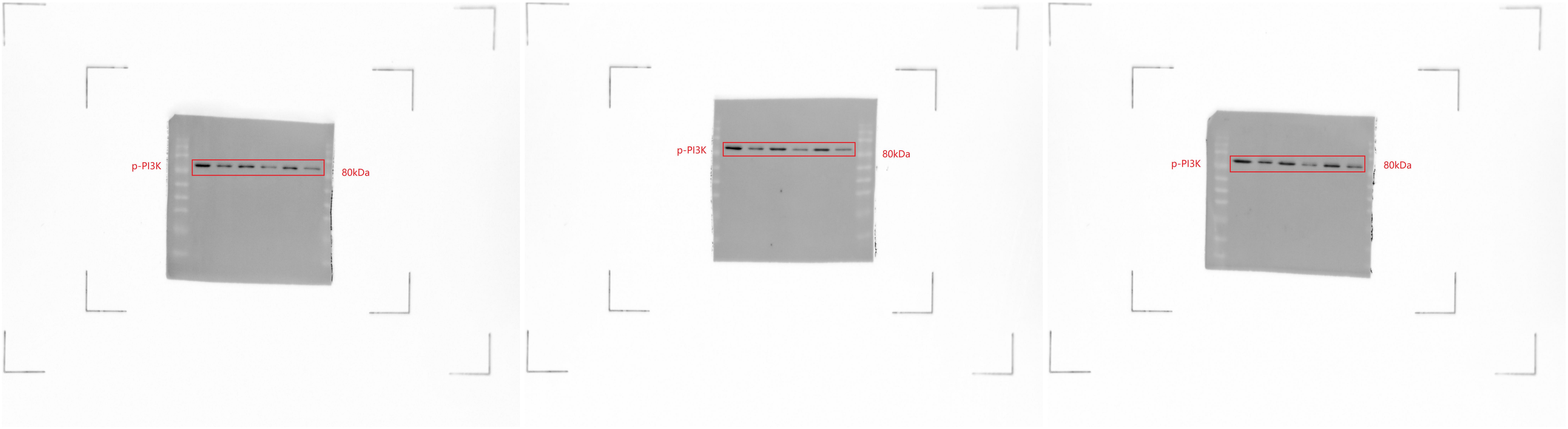

Figure 8B

The original blot of GAPDH in MEG-01 cells

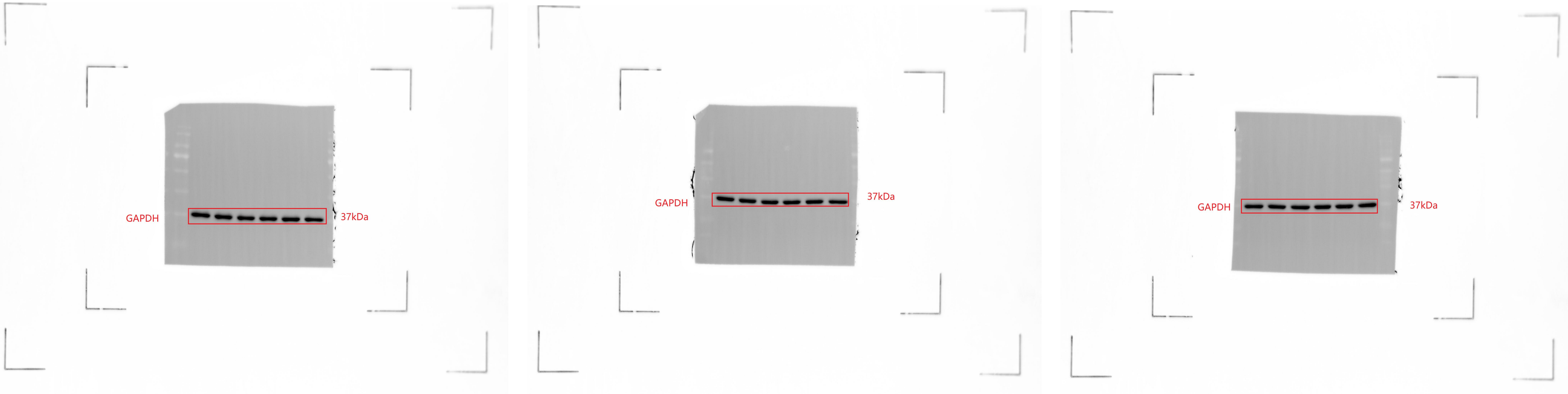

Figure 8B

The original blot of AKT in MEG-01 cells

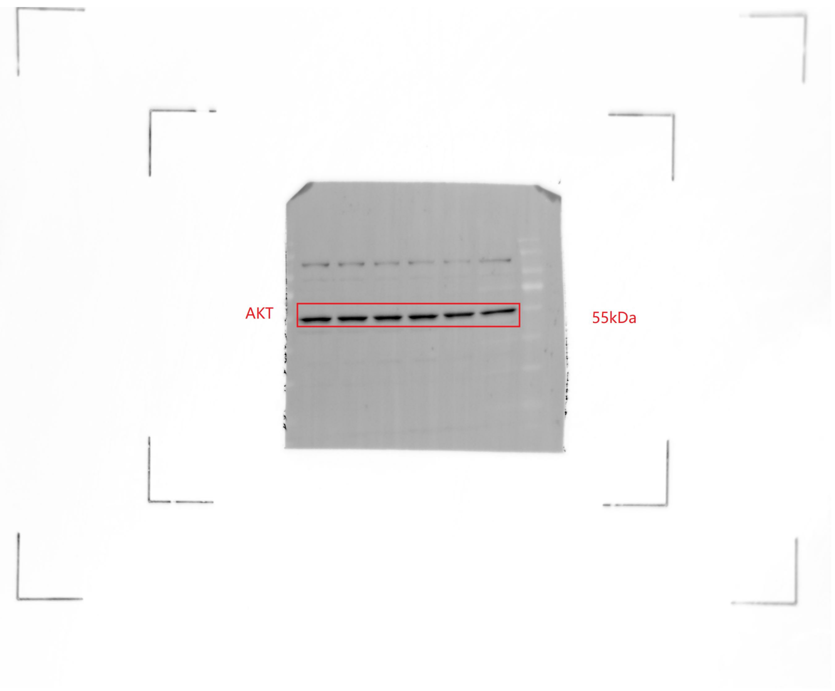

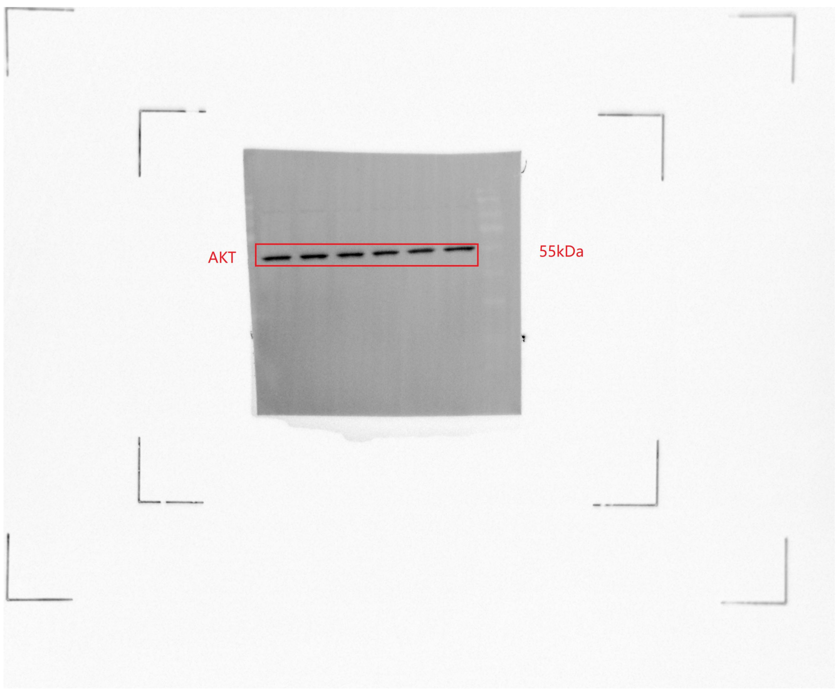

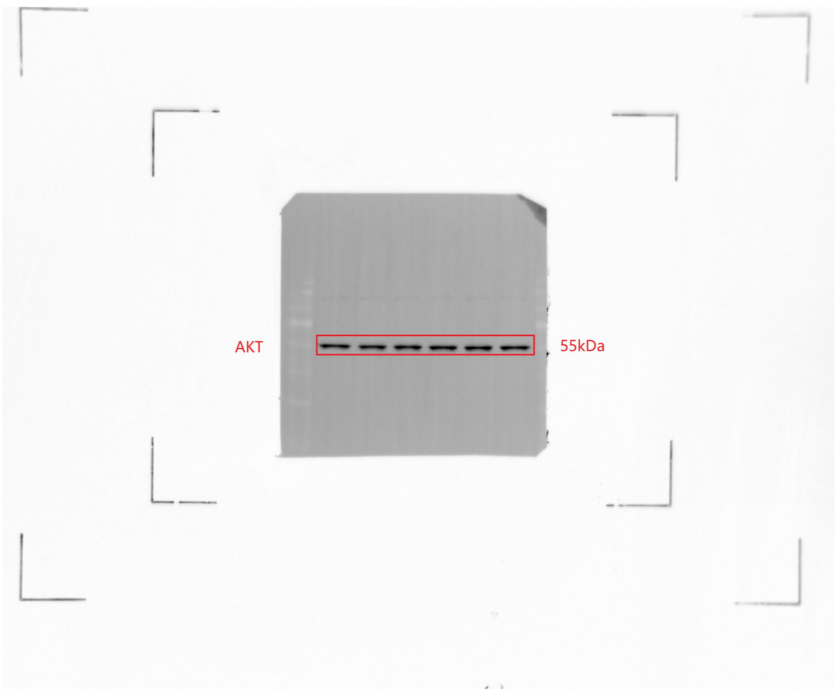

Figure 8B

The original blot of P-AKT in MEG-01 cells

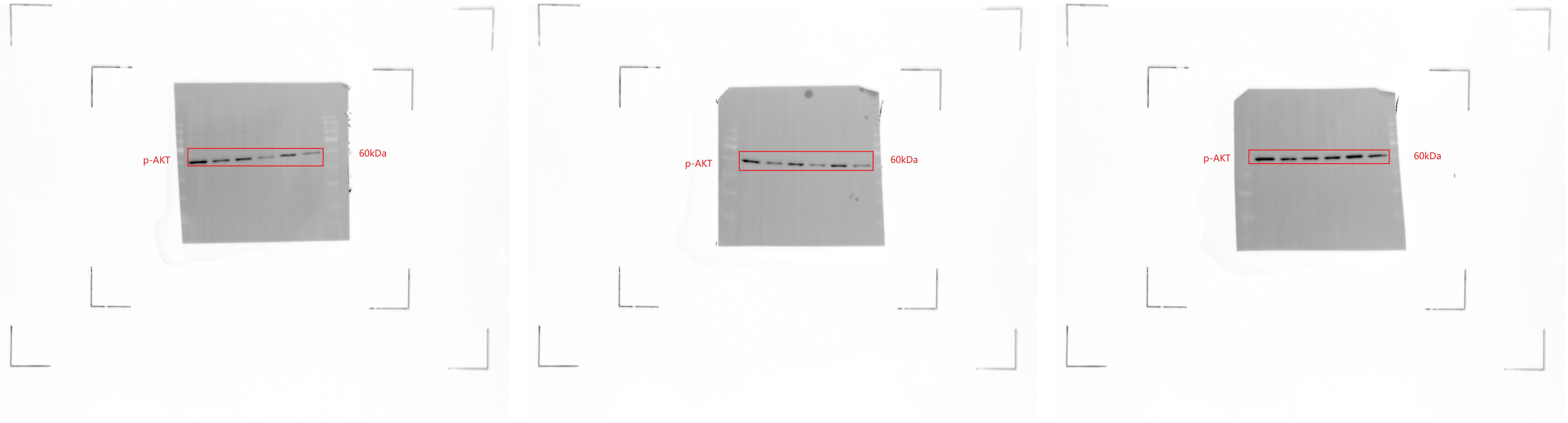

Figure 8B

The original blot of mTOR in MEG-01 cells

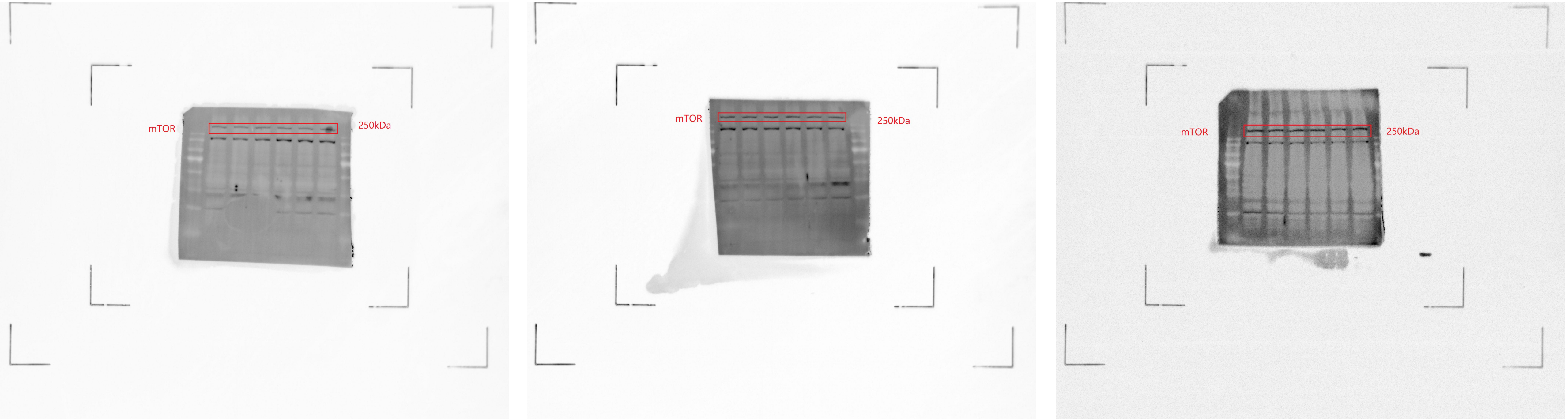

Figure 8B

The original blot of P-mTOR in MEG-01 cells

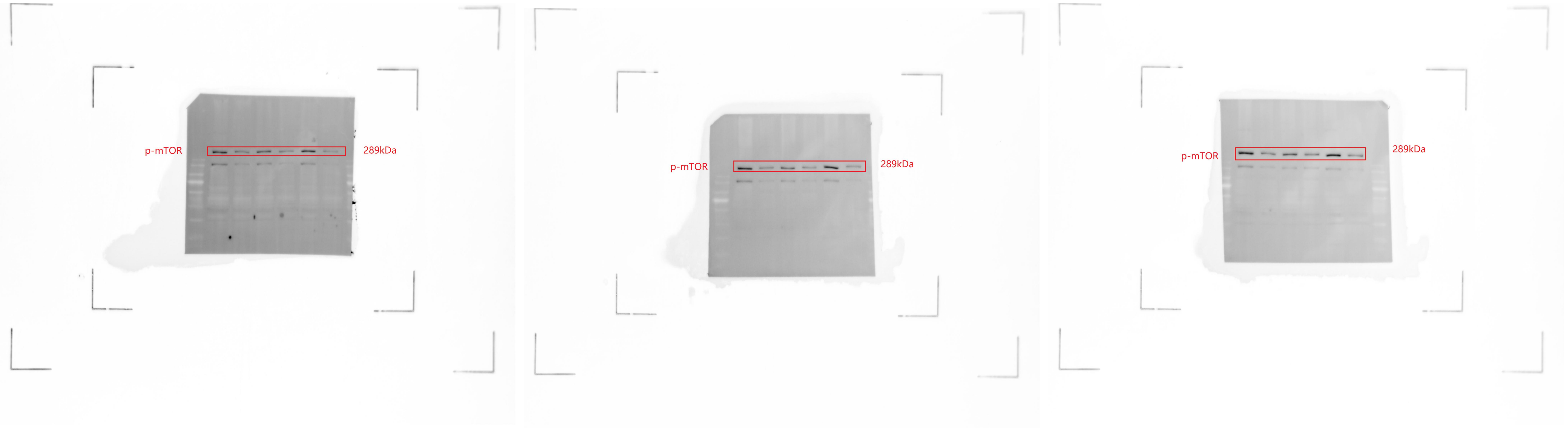

Figure 8B
The original blot of ERK in MEG-01 cells

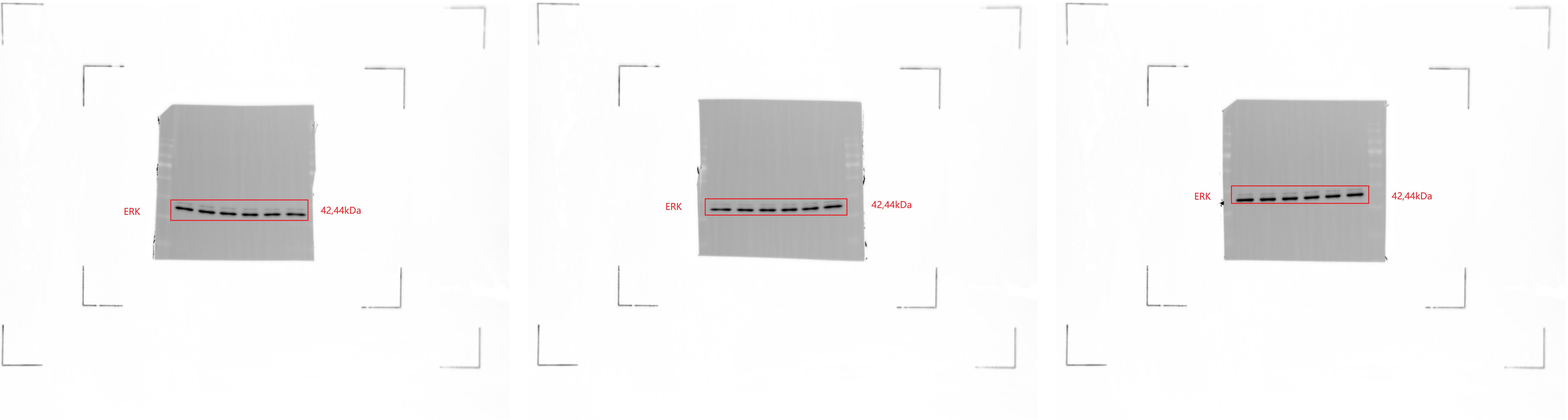

Figure 8B
The original blot of P-ERK in MEG-01 cells

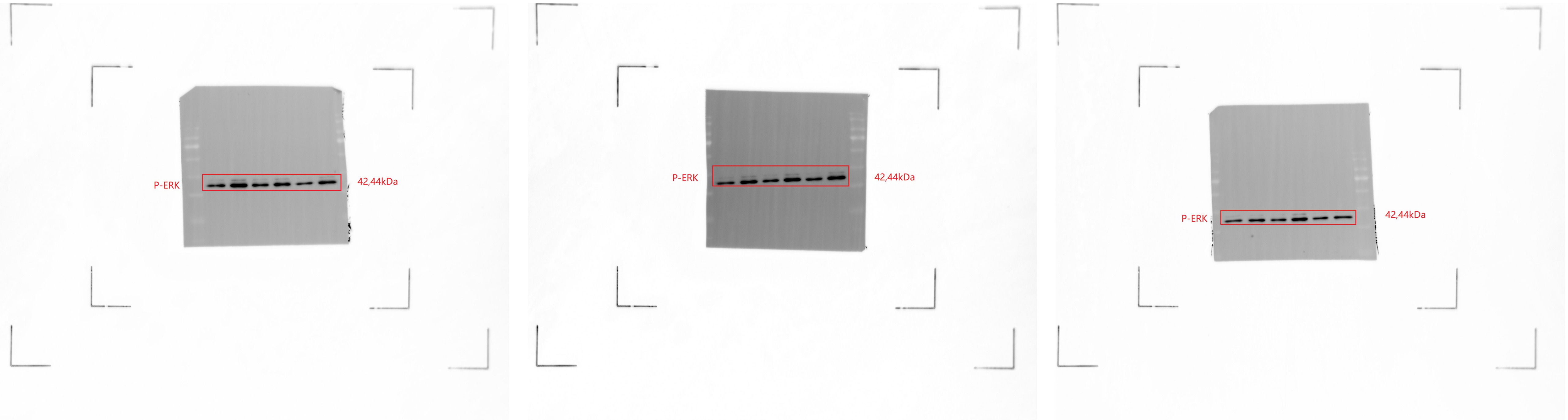

Figure 8B
The original blot of MAPK in MEG-01 cells

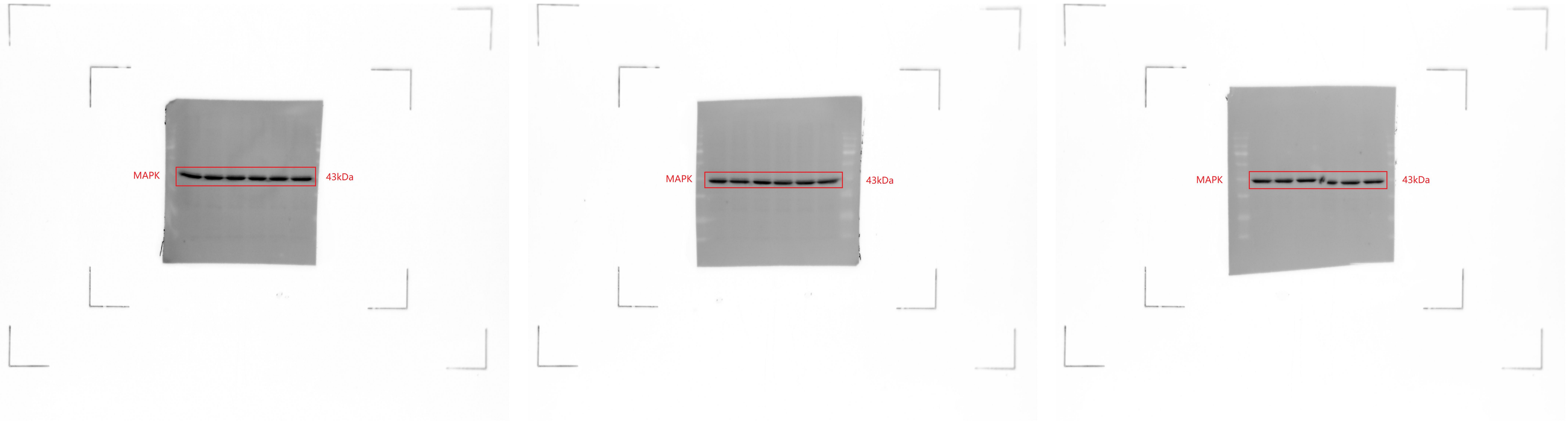

Supplement: Supplemental Information 2 [file peerj-11-16404-s002.pdf]
